# Variability in grain, flour, functional properties and protein profiling in newly developed wheat genotypes grown in temperate climate of Western Himalayas

**DOI:** 10.3389/fnut.2025.1604775

**Published:** 2025-07-15

**Authors:** Taha Mukhtar, Abida Jabeen, Syed Zameer Hussain, Raashid Ahmad Siddiqi, Shabir H. Wani, Anju Mahendru-Singh, Tawheed Amin, Quraazah A. Amin, Mohmad Sayeed Bhat

**Affiliations:** ^1^Division of Food Science and Technology, Sher-e-Kashmir University of Agricultural Sciences & Technology of Kashmir (SKUAST-K), Shalimar-Srinagar, J&K, India; ^2^Department of Food Technology, Eternal University, Baru-Sahib, HP, India; ^3^Mountain Research Centre for Field Crops, Sher-e-Kashmir University of Agricultural Sciences & Technology of Kashmir (SKUAST-K), Khudwani, Kulgam, J&K, India; ^4^Division of Genetics, Indian Agricultural Research Institute, New Delhi, India

**Keywords:** wheat genotypes, protein content, solvent retention capacity, falling number, SDS-PAGE

## Abstract

**Background:**

Since wheat varieties and breeding lines developed by plant breeders in the western Himalayan region (Kashmir) have received limited attention with respect to their physical and protein quality characterization, the focus of the study is to evaluate the diversity in grain, flour and the various protein fractions in newly released wheat genotypes grown in temperate regions of the Western Himalayas.

**Methods:**

The present study investigated the grain and flour quality in various newly developed advanced wheat genotypes, viz., KWQ-21-1, KWQ-21-2, KWQ-21-3, KWQ-21-4, SKW 374, KWQ-21-6, KWQ-21-7, and SKW 357, along with two released varieties (Shalimar Wheat-2 and Shalimar Wheat-3). These wheat genotypes were studied for their functional, structural and protein profiling characteristics as per the approved AACC and AOAC standard methods of analysis.

**Results:**

The various physical parameters varied significantly (*p* ≤ 0.05) between wheat genotypes. Protein content varied significantly from 8.06 to 11.39%. All the studied genotypes/varieties showed significant variations (*p* ≤ 0.05) with respect to proximate composition, gluten content, SDS sedimentation value, falling number and solvent capacities. Dry and wet gluten content varied from 8.78 to 13.2% and 23.27 to 33.87%, respectively. Wheat genotypes exhibited moderate SDS-sedimentation values and solvent retention values, indicating that the genotypes have moderate gluten strength and may be more suited for chapatti or biscuit making. A high degree of polymorphism in the intensity and number of bands in the molecular range of 30.7–48 kDa corresponding to the low molecular weight glutenin subunit (LMW-GS) /α-, β- and γ-gliadin region was observed in the wheat genotypes. High molecular weight (HMW) proteins varied from 76.5 to 111.5 kDa. Correlation results showed that the flour characteristics were related to each other, which can ultimately affect the end product utilization of these wheat genotypes.

## 1 Introduction

Wheat (*Triticum aestivum*) stands as one of the world's most extensively developed agricultural products, serving as the primary provider of phytonutrients, energy, vitamins, fiber, and protein for about 2.5 billion individuals. It provides the world's population with more energy, protein, and minor nutrients, including lipids, vitamins, fiber, and phytochemicals, than any other agricultural produce (1). The projected worldwide wheat production for the year 2024 was 797.3 million tons (2).

India is home to a large number of wheat cultivars that vary greatly in terms of their physicochemical, functional, and rheological characteristics, as reported by Kundu et al. (3). In India, wheat is a staple of the majority of the population and a major chunk of the population depends on it for its energy and protein requirements, as well as a source of fiber and, to some extent, a source of phytochemicals. India is a very diverse country with varying climatic conditions, due to which a large number of varieties with varying properties are constantly propagated and bred, exhibiting a variety of shapes, sizes, weights, and colors. It is essential to have a thorough understanding of the physical properties of wheat for operations such as sweeping, grading, dividing, storing, shipping, packaging, aeration, and milling yield. A number of factors influence the final product quality of wheat, which include protein concentration and quality in flour, wet and dry gluten content, and solvent retention capacities. Additionally, the protein fractions, such as albumins, globulins, α-, γ-, and ω-gliadins, high-molecular-weight glutenin subunit (HMW-GS), and low-molecular-weight glutenin subunit (LMW-GS), also play a significant role. These characteristics usually decide the quality of wheat flour and its end-product utilization.

Wheat quality is also impacted by a number of ecological factors, such as the chemical and physiological characteristics of the soil and geographical latitude. Enough sunlight, appropriate soil moisture, and a somewhat high temperature may all influence the quality of wheat (4). The environmental variables have an important effect on the accumulation of protein in wheat grain and processing quality (5). It is projected that the temperate climate in the Western Himalayas (Kashmir valley) would affect the amount and quality of wheat grain protein. Increasing attention is being directed toward the assessment of protein quality and quantity in wheat varieties and breeding lines cultivated under diverse agro-climatic conditions, particularly in light of global climate change, as both grain quality and yield are vital for ensuring human nutrition and food security. Asseng et al. (4) found that grain and protein yields are lower and more variable in most low-rainfall regions, thus further promoting the study of the wheat protein quantity as well as the quality of new wheat varieties/breeding lines because as the global weather pattern changes drastically, so does the amount of precipitation. The priority has also gradually shifted to the improvement of processing quality, primarily due to the increase in food diversity and market demand (6).

Since wheat varieties and breeding lines developed by plant breeders in the Western Himalayan region (Kashmir) have received limited attention with respect to their physical and protein quality characterization, bakers in this region often encounter challenges in obtaining flour of in-line quality for their products. There exists little to no comprehensive research on the protein fractions of newly developed wheat genotypes cultivated in this region, or there is just sporadic information available. Therefore, the objective of this study was to evaluate the diversity in grain, flour, and the various protein fractions in newly released wheat genotypes grown in temperate regions of the Western Himalayas (Kashmir).

## 2 Materials and methods

### 2.1 Plant materials and growth conditions

The experimental wheat trials were conducted during the growing Rabi season (crops are sown in late October to November and are harvested in May–June) of the years 2018 and 2019 at the Mountain Research Centre for Field Crops (MRCFC) in Khudwani (Anantnag-South Kashmir) with geographical coordinates 33°44′04.0″N and 75°05′13.3″E. The experimental material consisted of 101 wheat genotypes, including four check varieties—Shalimar Wheat-2, Shalimar Wheat-3, VL 907, and HS-562—obtained from CIMMYT-Mexico and ICAR-IIWBR Karnal exotic nurseries. A randomized block design (RCBD) was used for the field trials with a plot size of 1 x 1 m with six rows for each genotype, with three replications. The distance between the rows was 20 cm. The wheat samples were grown in clay loam soil under rainfed conditions without artificial irrigation. Fertilizer dose comprising nitrogen (N) (RDN) @120 kg N+ and phosphorus (P) (RDP) @ 60 kg P2O5 ha^−1^ was applied during different stages of crop growth. Fertilizers for N, P, and K were used in the form of urea, diammonium phosphate, and muriate of potash (K), respectively. Uniform recommended doses of K @ 30 kg K_2_O were also applied. Nitrogen was applied in three splits (basal at sowing, first top dose at maximum tillering, and second top dose at booting stage). Phosphorus was applied as a basal treatment. Standard crop management practices as per the package of practices were followed during the rest of the growing period. Mean weekly meteorological data for the duration of the experiments are presented in Supplementary Table S1. The yield and related morphological traits were recorded for all genotypes in each replication. Eight advanced wheat genotypes (KWQ-21-1, KWQ-21-2, KWQ-21-3, KWQ-21-4, SKW 374, KWQ-21-6, KWQ-21-7, and SKW 357) and two released wheat varieties (Shalimar Wheat-2 and Shalimar Wheat-3) developed at the Mountain Research Centre for Field Crops (MRCFC) in Khudwani, Sher-e-Kashmir University of Agricultural Sciences and Technology, Shalimar, Srinagar, India, were used for this study. First, the seeds were thoroughly cleansed to get rid of any extraneous objects, including broken and immature grains, dust, and stones. The tempering of grains was carried out as per AACC method 26-95.01 (7). The seeds were cleansed and tempered with ionized water until their moisture content reached 14% and left over to equilibrate at 4°C for 24 h to ensure proper tempering. The tempered kernels of all the genotypes/varieties were milled using a Brabender Quadrumat senior mill (Brabender GmbH, Germany) to produce white flour with a 72% rate of extraction. All the flour samples were collected and kept at −20°C in airtight jars until needed. Before use, each flour sample was defrosted at 25°C for 2 h.

### 2.2 Grain properties

Physical properties of all the wheat varieties were studied using a vernier caliper with a minimum 0.02 mm reading for measuring length, breadth, and thickness. Dimensional specifications, including equivalent diameter (Dm), L/W ratio, seed volume (V), surface area (A), aspect ratio (Ra), and sphericity (Φ), were also determined using the following equations as mentioned by Rani et al. (8), while the determination of gravimetric properties, such as test weight and thousand kernel weight, was carried out as per Amir et al. (9). Furthermore, the color profile of grains and flour with respect to L^*^, a^*^, and b^*^ values was assessed by utilizing a Tristimulus Colorimeter (Model SC-10, Sucolor, China), following the protocol outlined by Siddiqi et al. (10) with slight modifications.


(1) $$\begin{array}{l} \text{Equivalent diameter }\left(\text{Dm}\right)={\left(\text{LWT}\right)}^{1/3} \end{array}$$

(2) $$\begin{array}{l} \text{Seed volume }\left(\text{V}\right)\;=\;\frac{{\text{π B}}^{2}{\text{L}}^{2}}{6\left(2\text{L}-3\right)} \end{array}$$

(3) $$\begin{array}{l} \text{B}=\;{\left(\text{WT}\right)}^{1/2} \end{array}$$

(4) $$\begin{array}{l} \text{Surface area }\left(\text{S}\right)\;=\frac{{\text{π BL}}^{2}}{\left(2\text{L}-\text{B}\right)} \end{array}$$

(5) $$\begin{array}{l} \text{Aspect Ratio }\left(\text{Ra}\right)=\text{W}/\text{L} \end{array}$$

(6) $$\begin{array}{l} \text{Sphericity }\left(\text{Φ}\right)\;=\;\frac{{\left(\text{LWT}\right)}^{1/3}}{\text{L}}\;\times \;100 \end{array}$$

(7) $$\begin{array}{l} \text{Hueangle }\left(\text{H}\right)=\tan^{-1\;}\left({\text{b}}^{*}/{\text{a}}^{*}\right) \end{array}$$

(8) $$\begin{array}{l} \text{Chroma }\left({\text{C}}^{*}\right)={\left(\;{{\text{a}}^{*}}^{2}+{{\text{b}}^{*}}^{2\;}\right)}^{0.5}\; \end{array}$$

where W = width; L = length; T = thickness of grains.

### 2.3 Physicochemical properties of wheat flour samples

#### 2.3.1 Proximate composition

Moisture, protein, fat, ash, and crude fiber were estimated following the AACC method (7). Carbohydrate was calculated using the difference method. The energy values were determined by multiplying the carbohydrate and protein content by 4 kcal/g and the fat content by 9 kcal/g (10).

#### 2.3.2 Gluten content

Wet and dry gluten was analyzed by approved AACC method 38-10.01 (7). The dough was prepared by thoroughly mixing approximately 12 ml of water with 25 g of flour in a porcelain dish. The dough was shaped into a spherical mass and immersed in a beaker containing water for a minimum duration of 60 min. Following the designated resting period, the dough was gently kneaded under running tap water over a 75-mm sieve until the rinsing water transitioned from milky to clear, indicating the removal of starch and other water-soluble components. The resulting sticky or dark-colored mass was subsequently immersed in a beaker containing water and left undisturbed for an additional 60 min. Subsequently, the gluten was manually compressed between both hands to expel excess water, shaped into a spherical mass, and weighed to determine the wet gluten content. The wet gluten was subsequently dried in a hot air oven at 110°C for 24 h, cooled in a desiccator to ambient temperature, and then weighed to determine the dry gluten content. The wet and dry gluten was then determined using the following formulas.


(9) $$\begin{array}{l} \text{Wet gluten }\left(\text{\%}\right)=\frac{\text{Weight of wet gluten }\left(\text{g}\right)}{\text{Weight of the sample }\left(\text{g}\right)}\times 100\; \end{array}$$

(10) $$\begin{array}{l} \text{Dry gluten }\left(\text{\%}\right)=\frac{\text{Weight of dry gluten }\left(\text{g}\right)}{\text{Weight of the sample }\left(\text{g}\right)}\times 100 \end{array}$$

### 2.4 Flour performance properties

#### 2.4.1 Solvent retention capacity

Solvent retention capacity (SRC) was computed following the methodology outlined by Holkovicova et al. (11), with slight adjustments. One gram of flour was individually suspended in standard solutions (5 ml of deionized water, 5% lactic acid, 5% sodium carbonate, and 50% sucrose), and the flour suspension was allowed to hydrate and mix properly at 150 rpm for 15 min on a horizontal shaking incubator (Labtech India). The suspended samples were centrifuged at 1,000 × g for 15 min at 25°C. The supernatant was carefully removed; the excess liquid was drained from the sample tubes by tilting both of them by 90 degrees for 15 min. After draining, the tubes were weighed. The SRC values were computed using the formula:


(11) $$\begin{array}{l} \text{SRC }\left(\text{g}/100\text{ g}\right)=\frac{\text{wet pellet }\left(\text{g}\right)}{\text{flour }\left(\text{g}\right)}-1\\ \;\times \frac{86}{100-\text{Flour moisture }\left(\frac{\text{g}}{100\text{ g}}\right)}\;\times 100 \end{array}$$

#### 2.4.2 Gluten performance index

The Gluten Performance Index (GPI) was calculated by following the formula outlined by Yang et al. (12) using the SRC data,


(12) $$\begin{array}{l} \text{GPI}=\frac{\text{Lactic acid SRC }}{\text{Sodium Carbonate SRC}+\text{Sucrose SRC}} \end{array}$$

#### 2.4.3 Alkaline water retention capacity

Alkaline water retention capacity (AWRC) was calculated according to the procedure of Omran et al. (13). AWRC value for each flour sample was calculated using the specified formula.


(13) $$\begin{array}{l} \text{AWRC}\left(\frac{\text{g}}{100\text{ g}}\right)=\text{wet}\frac{\text{pellet }\left(\text{g}\right)}{\text{flour }\left(\text{g}\right)}-1\times \;\frac{86}{100}-\text{flour moisture }\\ \;(\frac{\text{g}}{100\text{ g}})\times 100 \end{array}$$

### 2.5 Functional properties of flour

#### 2.5.1 SDS-sedimentation value

The sodium dodecyl sulfate (SDS) sedimentation value was determined by following the method defined by Liaquat et al. (14) with minor modifications. Five grams of flour were added to a 50-ml measuring cylinder with a stopper and mixed vigorously for 15 seconds. After 2 and 4 min, the mixture was again thoroughly stirred for 15 seconds each time. Immediately afterward, 50 ml of freshly prepared SDS–lactic acid reagent (made by dissolving 2 g of SDS in 100 ml of water and adding 2 ml of a diluted lactic acid stock solution prepared by mixing one-part lactic acid with eight parts water by volume) was added. The contents were mixed by inverting the cylinder four times and the timer was reset. Inversions were repeated four times at 2, 4, and 6 min, with the timer restarted each time. The mixture was then allowed to settle undisturbed for 40 min, after which the sedimentation measurements were taken.

#### 2.5.2 Water and oil absorption capacities

Water absorption capacity (WAC) and oil absorption capacity (OAC) were determined using the procedure of Verem et al. (15) with slight modifications. One gram of the flour was combined with 10 ml of either refined soybean oil or distilled water, vortexed for 10 seconds every 5 min for a total time spanning 30 min, and then centrifuged at 2,500 × g (for 30 min at 20°C). After centrifugation, the water-based supernatant or transparent oil was removed, the test tubes were turned upside down, and they were left to drain on a paper towel for 5 min. The residue was weighed to compute the WAC and OACs using the following formula:


(14) $$\begin{array}{l} \text{WAC }\left(\text{\%}\right)=\frac{\text{W}2-\text{ W}1}{\text{W}}\times 100 \end{array}$$

W_2_= final weight of the tube after water decanted off, W_1_ = initial tube weight, and W= weight of the sample.


(15) $$\begin{array}{l} \text{OAC }\left(\text{\%}\right)=\frac{\text{W}4-\text{W}3\;}{\text{W}3}\;\times 100 \end{array}$$

where W_3_ is the initial flour mass and W_4_ is the flour mass after immersion in oil and centrifugation.

#### 2.5.3 Swelling capacity

The method described by Alam et al. (16) was used to assess the swelling capacity. A sample up to the 10 ml mark was put into the 100-ml graduated cylinder. Deionized water was added until the total volume reached 50 ml. The opening of the graduated cylinder was sealed tightly, and the contents were mixed by carefully turning the cylinder upside down. Two minutes later, the suspension was reversed again and left to settle for eight more minutes. At the end of the 8-min interval, the volume displaced by the sample was measured.

#### 2.5.4 Emulsion capacity and emulsion stability

The emulsion capacity (EC) and emulsion stability (ES) were determined by the method described by Twinomuhwezi et al. (17). One gram of wheat flour, 10 ml of deionized water, and 10 ml of vegetable oil were mixed together in a calibrated centrifuge tube. The mixture was then subjected to vortex homogenization at 7,000 rpm for 5 min to ensure the formation of a proper emulsion. This homogenization step facilitates the dispersion of oil droplets within the aqueous phase, forming a temporary emulsion. After homogenization, the emulsion underwent a 5-min, 2,000 × g centrifugation. The emulsion capacity (in %) was determined by dividing the height of the emulsion layer by the height of the mixture as a whole. Using a calibrated centrifuge tube, the emulsion was heated to 80°C for 30 min in a water bath, cooled for 15 min under running water, and then centrifuged for 15 min at 2,000 × g to assess the emulsion stability of the flour samples. The emulsion stability was determined by calculating the percentage difference between the height of the emulsified layer and the height of the mixture as a whole.


(16) $$\begin{array}{l} \text{Emulsion capacity }\left(\text{\%}\right)=\frac{\text{ Height of emulsion layer }\left(\text{ml}\right)}{\text{Total height }\left(\text{ml}\right)}\;\times 100\; \end{array}$$

(17) $$\begin{array}{l} \text{Emulsion stability }\left(\text{\%}\right)=\\ \frac{\text{ Height of emulsion layer after heating }\left(\text{ml}\right)}{\text{Height before }\left(\text{ml}\right)}\;\times 100 \end{array}$$

#### 2.5.6 Falling number

The falling number (FN) of the flour samples was determined using the Falling Number 1305 system (Perkin Elmer Inc., Sweden). The measurement was conducted in accordance with the AACC Approved Method 56–81.03 (7). Seven grams of sample flour was mixed with 25 ml of distilled water in an FN tube. The tube was shaken vigorously for 3 seconds manually. The tube was fitted with a viscometer-stirrer and then inserted into the falling number machine. The falling number tube was placed in the hot water bath for 5 seconds and stirred for 55 seconds subsequently; the machine recorded the duration required for the stirrer to descend from the tube's top to its bottom. The FN reading was determined by adding the 5 seconds the sample stood in boiling water, the 55 seconds of stirring, and the time it took for the stirrer to descend.

### 2.6 SDS-PAGE of total flour proteins

SDS-PAGE of flour was carried out following the procedure of Siddiqi et al. (10). Hexane was used in a 1:4 ratio to defatinate the flour, and the process was repeated three times. Fifty milligrams (50 mg) of defatted wheat flour were weighed and placed into sterilized 1.5 ml Eppendorf tubes. Subsequently, 1 ml of 2X Laemmli sample buffer solution (pH 6.8, comprising 62.5 mM Tris–HCl, 2% SDS, 5% ß-mercaptoethanol, 25% glycerol, and 0.01% bromophenol blue) was added immediately to the flour. The flour samples were initially vortexed to achieve a uniform suspension. Following this, the tubes were subjected to horizontal agitation in an orbital shaker at 151 rpm for 1 h at 45°C to facilitate thorough mixing. Subsequently, the samples were heated in a water bath at 100°C for 5 min. Finally, the mixtures were centrifuged at 11,000 × g for 15 min. The supernatant (10 μl) was loaded in each well (Mini-Protean Tetra Cell, Bio-Rad Laboratories, Hercules, USA). Proteins were separated using 4% stacking and 12% resolving gel. The gels were run at 25 mA. The protein marker (HiMedia Pre-stained Protein Ladder from Maharashtra, India) was used to calculate the molecular weights of the flour polypeptides. After destaining, the gels were photographed using the i-Bright™ CL1500 Imaging System (Invitrogen, Thermo Fisher Scientific) and quantified using the Bio-Rad EZ Imager (Bio-Rad Laboratories, Hercules, CA, USA). The gels were classified into different protein subgroups following the procedure of Siddiqi et al. (10). The proportion of each band was calculated by standardizing its intensity relative to the total intensity of all bands within a specific lane, setting the total to 100%. The area of each subunit in relation to the total extractable proteins was then used to calculate the percentage of each flour protein in the various wheat varieties. SDS-PAGE analysis was carried out two times using aliquots of the same sample.

### 2.7 Fourier transform infrared spectroscopy of wheat flour samples

Fourier transform infrared spectroscopy (FTIR) (Spectrum Two, PerkinElmer) was performed over a defined frequency range from 400 cm^−1^ to 4,000 cm^−1^ at a resolution of 4 cm^−1^ and calculated over 64 scans. The wheat flour was placed on an ATR crystal, which is made up of zinc selenide. The sample's single-beam spectrum was acquired to display the spectrum in absorbance units. In between studies, the ATR crystal was completely cleaned using methanol.

### 2.8 Statistical analysis

The experimental results were reported as the mean ± standard deviation from three replicates. Duncan's test was used to analyze differences between mean values at a significance level of *p* ≤ 0.05. Pearson's correlation coefficient (significance levels at *p* ≤ 0.05 and *p* ≤ 0.01) was performed using SPSS version 16.0 (SPSS Inc., Chicago, IL, USA) to determine the relationship between different parameters.

## 3 Results and discussion

### 3.1 Physical characteristics of wheat grains

The shape and size of grains are critical physical attributes that influence quality assessment, grain screening processes, and calculations related to heat and mass transfer. These characteristics affect the efficiency of separating foreign materials and are essential parameters in designing and optimizing thermal processes in grain handling and storage systems (8). Length, width, thickness, and equivalent diameter are indicators of size. The physical attributes of the wheat grains are presented in Table 1. The length, width, and thickness of wheat grains were observed in the ranges of 6.11 to 7.69 mm, 2.20 to 2.80 mm, and 2.97 to 3.80 mm, respectively. The length of KWQ-21-1 was significantly (*p* ≤ 0.05) higher than SKW 357. The width varied between 2.20 mm in KWQ-21-7 and 2.80 mm in SKW 374. The thickness was recorded as highest for KWQ-21-2 (3.80 mm) and lowest for KWQ-21-7 (2.97 mm). Shalimar wheat variety 3 had the highest length-to-breadth (L/B) ratio at 2.89, while SKW 374 had the lowest ratio at 2.25. The equivalent diameter exhibited a statistically significant variation (*p* ≤ 0.05), ranging from 3.50 to 4.16 mm.

**Table 1:** Physical and gravimetric characteristics of the newly developed wheat genotypes grown in the Western Himalayas.

**Variety**	**Length (mm)**	**Width/breadth (mm)**	**Thickness (mm)**	**Aspect ratio**	**Equivalent diameter (mm)**	**Sphericity (%)**	**Seed volume (mm^3^)**	**Surface area (mm^2^)**	**L/B ratio**	**Thousand kernel weight (g)**	**Test weight (kg/hl)**
KWQ-21-1	7.69 ± 0.11^A^	2.70 ± 0.11^A^	3.50 ± 0.17^AB^	0.36 ± 0.06^A^	4.16 ± 0.03^A^	54.22 ± 0.06^G^	23.56 ± 0.06^A^	46.3 ± 0.06^A^	2.84 ± 0.06^AB^	59.68 ± 0.12^A^	80.50 ± 0.15^E^
KWQ-21-2	6.34 ± 0.12^E^	2.73 ± 0.08^A^	3.80 ± 0.11^A^	0.42 ± 0.09^A^	4.11 ± 0.04^A^	63.56 ± 0.06^A^	22.53 ± 0.06^C^	42.93 ± 0.04^C^	2.32 ± 0.06^D^	56.54 ± 0.17^B^	80.54 ± 0.13^E^
KWQ-21-3	7.05 ± 0.04^BC^	2.80 ± 0.05^A^	3.20 ± 0.11^BC^	0.37 ± 0.09^A^	3.91 ± 0.03^AB^	56.31 ± 0.06^E^	20.80 ± 0.06^E^	41.82 ± 0.06^D^	2.50 ± 0.06^CD^	59.54 ± 0.18^A^	83.47 ± 0.15^B^
KWQ-21-4	6.22 ± 0.03^E^	2.60 ± 0.11^AB^	3.20 ± 0.05^BC^	0.41 ± 0.06^A^	3.72 ± 0.02^BCD^	59.8 ± 0.06^C^	17.78 ± 0.06^H^	36.59 ± 0.058^H^	2.30 ± 0.06^D^	47.72 ± 0.14^F^	85.47 ± 0.16^A^
SKW 374	6.30 ± 0.05^E^	2.80 ± 0.05^A^	3.56 ± 0.14^AB^	0.43 ± 0.05^A^	3.95 ± 0.02^AB^	63.09 ± 0.04^B^	21.46 ± 0.06^D^	41.54 ± 0.06^E^	2.25 ± 0.06^D^	54.60 ± 0.14^C^	82.59 ± 0.18^C^
KWQ-21-6	7.13 ± 0.03^B^	2.76 ± 0.08^A^	3.53 ± 0.14^AB^	0.38 ± 0.06^A^	4.11 ± 0.05^A^	57.64 ± 0.06^D^	23.05 ± 0.06^B^	44.70 ± 0.06^B^	2.58 ± 0.06^ABCD^	51.49 ± 0.28^E^	74.57 ± 0.19^G^
KWQ-21-7	6.81 ± 0.06^CD^	2.20 ± 0.05^C^	2.97 ± 0.01^C^	0.33 ± 0.06^A^	3.5 ± 0.03^D^	51.39 ± 0.06^H^	14.86 ± 0.06^J^	33.54 ± 0.06^I^	2.80 ± 0.06^ABC^	52.49 ± 0.21^D^	79.06 ± 0.07^F^
Shalimar Wheat-2	6.73 ± 0.12^D^	2.66 ± 0.08^AB^	3.00 ± 0.11^C^	0.38 ± 0.06^A^	3.77 ± 0.05^BC^	56.09 ± 0.04^E^	18.067 ± 0.03^G^	37.69 ± 0.06^G^	2.53 ± 0.06^BCD^	45.50 ± 0.25^G^	81.52 ± 0.16^D^
SKW 357	6.11 ± 0.06^E^	2.20 ± 0.11^C^	3.30 ± 0.11^BC^	0.36 ± 0.06^A^	3.53 ± 0.06^CD^	57.77 ± 0.06^D^	15.327 ± 0.06^I^	33.08 ± 0.04^J^	2.77 ± 0.06^ABC^	45.53 ± 0.16^H^	82.59 ± 0.15^C^
Shalimar Wheat-3	6.94 ± 0.07^BCD^	2.40 ± 0.05^BC^	3.40 ± 0.11^B^	0.34 ± 0.06^A^	3.84 ± 0.06^B^	55.61 ± 0.06^F^	18.81 ± 0.06^F^	39.083 ± 0.05^F^	2.89 ± 0.06^A^	45.56 ± 0.25^G^	85.39 ± 0.12^A^

Data are presented as the mean ± SD. Means with different superscripts in columns differ significantly (*p* ≤ 0.05). *n* = 3.

Shape is usually expressed through metrics such as aspect ratio and sphericity. The extent to which an object mimics the shape of a sphere of equal volume is defined by its sphericity. The aspect ratio varied between 0.33 and 0.43. SKW 374 had the highest aspect ratio, while KWQ-21-7 had the lowest. The sphericity of grains ranged from 51.39% to 63.56%. KWQ-21-7 exhibited the minimum sphericity among the wheat types, whereas KWQ-21-2 exhibited the maximum. The sphericity values indicate that wheat seeds are relatively elongated and exhibit a higher tendency to slide rather than roll. Such a characteristic plays a key role in the development of dehullers, hoppers, and related processing equipment (3).

The seed volume of grains varied significantly (*p* ≤ 0.05) between 14.86 mm^3^ and 23.56 mm^3^. The wheat genotype KWQ-21-1 had the highest and KWQ-21-7 had the lowest seed volume. The surface area of the grain ranged between 33.08 and 46.3 mm^2^, with the highest in KWQ-21-1 and the lowest in SKW 357. Significant variability (*p* ≤ 0.05) was observed among the wheat genotypes for thousand kernel weight (TKW), with values ranging from 45.50 to 59.68 g. The highest thousand kernel weight (TKW) was observed in the genotype KWQ-21-1, while Shalimar Wheat-2 recorded the lowest. Thousand kernel weight (TKW) serves as a key determinant of both grain quality and milling or processing efficiency. Grains that are longer, plumper, and structurally sound generally contribute to higher TKW values, which are often associated with superior end-use quality, including better flour extraction rates and improved baking performance. Siddiqi et al. (10) and Rani et al. (8) previously reported the physical characteristics of 20 wheat cultivars from North India, with grain length ranging from 6.29 to 7.59 mm, width from 3.19 to 3.69 mm, and thickness from 2.74 to 3.39 mm. The length-to-breadth (L/B) ratio was reported between 1.78 and 2.15, while the equivalent diameter varied from 4.00 to 4.30 mm. Sphericity ranged from 56.31% to 64.67%, and the aspect ratio ranged from 0.46 to 0.56. Surface area and seed volume were observed in the ranges of 42.58–50.38 mm^2^ and 21.75–27.35 mm^3^, respectively. The thousand kernel weight (TKW) was reported to range from 33.05 to 51.26 g. The findings of our study closely align with previously reported results, with slight variations likely attributable to the use of different wheat genotypes and variations in cultivation practices. A significant variation (*p* ≤ 0.05) in test weight was observed among the genotypes, ranging from 74.57 kg/hl in KWQ-21-6 to 85.47 kg/hl in KWQ-21-4. Several studies have established that test weight is significantly influenced by environmental conditions, particularly nutrient availability and fertilizer management. Our findings are in agreement with those of Amir et al. (9), who reported test weights ranging from 69.25 to 80.35 kg/hl across five Pakistani wheat varieties.

### 3.2 Grain color

L*, a*, and b* values of grains of different wheat genotypes varied significantly (*p* ≤ 0.05) from 39.72 to 45.89, 8.85 to 10.71, and 20.15 to 25.34, respectively (Table 2). The highest L* value was observed for Shalimar Wheat-3, while the lowest value was observed for KWQ-21-3. The highest a* value was observed for SKW 374, while KWQ-21-1 showed lower values than other genotypes. The highest b* value was recorded for Shalimar Wheat-3, while the lowest value was recorded for KWQ-21-1. The Hue angle (H°) and Chroma (C*) values of grains showed significant variation (*p* ≤ 0.05), ranging from 63.88° to 67.29° and from 21.89 to 27.49, respectively (Table 2). The highest Hue angle (H°) value was observed for Shalimar Wheat-3 and the lowest value for KWQ-21-3. The highest Chroma (C*) value was found for Shalimar Wheat-3 and the lowest for KWQ-21-1, following a similar trend to b* values. Shalimar Wheat-3 demonstrated significantly higher lightness, as reflected by its elevated L^*^ value in the color scale, indicating a lighter seed coat than the other genotypes evaluated. Garg et al. (18) previously reported the color parameters of wheat grains, with L^*^ values ranging from 35.2 to 58.9, a^*^ values from 1.2 to 10.1, and b^*^ values from 11.5 to 27.4. The corresponding Chroma (C^*^) values ranged from 12.6 to 28.6, while the Hue angle (H°) varied between 58.8° and 85.0°. Katyal et al. (19) also investigated the color attributes of eight Indian wheat varieties, reporting L^*^ values ranging from 52.03 to 58.07, a^*^ values from 5.81 to 7.07, and b^*^ values from 16.63 to 20.13. These findings are closely aligned with the results obtained in this study, indicating comparable grain color characteristics. The coloration of wheat grains is primarily attributed to the presence of pigments such as carotenoids, flavonoids, anthocyanins, and various phenolic compounds. Variation in grain color among wheat varieties is influenced by the differential accumulation and distribution of pigment-related compounds, which are regulated by multiple factors, including soil composition, irrigation practices, climatic conditions, fertilizer application, and the genetic makeup of the cultivars (20).

**Table 2:** CIE color parameters of the grains and flour of newly developed wheat genotypes grown in the Western Himalayas.

**Variety**	**Color profile of wheat kernel**					**Color profile of wheat flour**				
	*L* ^*^	*a* ^*^	*b* ^*^	**Chroma**	**Hue**	*L* ^*^	*a* ^*^	*b* ^*^	**Chroma**	**Hue**
KWQ-21-1	39.74 ± 0.05^E^	8.85 ± 0.02^F^	20.15 ± 0.02^H^	21.89 ± 0.03^I^	66.21 ± 0.05^C^	73.45 ± 0.41^D^	3.31 ± 0.04^B^	10.44 ± 0.05^G^	10.98 ± 0.05^G^	71.90 ± 0.04^G^
KWQ-21-2	42.72 ± 0.08^D^	9.33 ± 0.03^E^	21.53 ± 0.05^G^	23.45 ± 0.05^H^	66.69 ± 0.05^B^	66.83 ± 0.57^G^	2.87 ± 0.02^D^	12.62 ± 0.09^D^	12.82 ± 0.05^D^	76.77 ± 0.06^A^
KWQ-21-3	39.72 ± 0.11^E^	10.55 ± 0.03^AB^	21.52 ± 0.02^G^	23.85 ± 0.02^G^	63.88 ± 0.06^F^	79.25 ± 0.05^B^	3.53 ± 0.05^A^	13.08 ± 0.05^B^	13.55 ± 0.05^B^	76.39 ± 0.02^B^
KWQ-21-4	43.83 ± 0.08^C^	10.36 ± 0.03^C^	23.25 ± 0.04^E^	25.45 ± 0.04^E^	65.84 ± 0.05^D^	67.96 ± 0.11^F^	3.01 ± 0.03^CD^	11.49 ± 0.05^F^	11.77 ± 0.06^F^	75.32 ± 0.05^D^
SKW 374	45.13 ± 0.05^AB^	10.71 ± 0.04^A^	24.15 ± 0.02^C^	26.51 ± 0.02^C^	66.11 ± 0.04^CD^	72.50 ± 0.08^E^	3.15 ± 0.03^BC^	12.12 ± 0.06^E^	12.51 ± 0.05^E^	75.61 ± 0.04^C^
KWQ-21-6	44.32 ± 0.03^BC^	10.36 ± 0.03^C^	21.50 ± 0.03^G^	23.75 ± 0.05^G^	64.32 ± 0.05^E^	79.13 ± 0.07^B^	3.48 ± 0.05^A^	13.17 ± 0.05^B^	13.62 ± 0.05^B^	75.26 ± 0.06^D^
KWQ-21-7	45.14 ± 0.60^AB^	9.56 ± 0.03^D^	22.63 ± 0.02^F^	24.60 ± 0.02^F^	67.22 ± 0.05^A^	78.92 ± 0.04^B^	3.31 ± 0.08^B^	12.69 ± 0.07^CD^	13.14 ± 0.05^C^	74.85 ± 0.07^E^
Shalimar Wheat-2	45.32 ± 0.08^AB^	10.56 ± 0.03^AB^	25.13 ± 0.05^B^	27.17 ± 0.05^B^	67.13 ± 0.05^A^	74.89 ± 0.05^C^	3.16 ± 0.05^BC^	13.12 ± 0.05^B^	13.58 ± 0.05^B^	76.45 ± 0.08^B^
SKW 357	43.52 ± 0.05^CD^	10.45 ± 0.03^BC^	23.76 ± 0.05^D^	25.84 ± 0.05^D^	66.31 ± 0.06^C^	80.16 ± 0.06^A^	3.54 ± 0.07^A^	12.80 ± 0.08^C^	13.28 ± 0.05^C^	74.42 ± 0.03^F^
Shalimar Wheat-3	45.89 ± 0.05^A^	10.59 ± 0.02^AB^	25.34 ± 0.03^A^	27.49 ± 0.04^A^	67.29 ± 0.05^A^	78.62 ± 0.05^B^	3.50 ± 0.05^A^	15.03 ± 0.05^A^	15.43 ± 0.05^A^	76.86 ± 0.05^A^

Data are presented as the mean ± SD. Means with different superscripts in columns differ significantly (*p* ≤ 0.05). *n* = 3.

### 3.3 Physicochemical properties of wheat flour samples

#### 3.3.1 Proximate composition

The results related to the proximate composition of flour obtained from selected wheat genotypes had significant (*p* ≤ 0.05) differences (Table 3). The moisture content of wheat flour ranged from 11.37% to 13.54%. The protein content of flour of different wheat genotypes varied significantly (*p* ≤ 0.05) and was found in the range of 8.0% to 11.39%. KWQ-21-1 had higher protein content (11.39%), whereas SKW357 (8.06%) had lower protein content. The fat content of different wheat flours was noted to be from 0.13% to 1.02%. SKW357 significantly had the highest fat content, whereas KWQ-21-2 had the lowest fat content. The ash content of wheat flour ranged from 0.11% to 0.46%. The percentage of crude fiber varied from 1.11% to 3.32%. The carbohydrate content of wheat flour ranged from 73.17% to 76.72%. The carbohydrate content was highest in Shalimar Wheat-2 (76.72%) and lowest in KWQ-21-1 (73.17%). The energy values varied significantly (*p* ≤ 0.05) from 337.47 (KWQ-21-6) to 346.73 (SKW 357) kcal/100 g. Memon et al. (21) previously reported moisture, protein, fat, ash, crude fiber, carbohydrates, and energy values of 7.13%−7.61%, 10.9%−11.8%, 0.12%−0.25%, 2.10%−2.77%, 0.26%−0.28%, 78.4%−79.7%, and 358.99–363 kcal/100 g, respectively, for three Pakistani wheat varieties. Siddiqi et al. (10) also previously analyzed the proximate composition of 14 wheat cultivars cultivated across diverse agro-climatic regions of North India: moisture (8.67%−11.46%), protein (9.32%−12.60%), fat (0.91%−1.51%), ash (0.41%−1.08%), crude fiber (0.08%−0.26%), carbohydrates (72.23%−79.35%), and energy value (352.23–368.11 kcal/100 g). The observed variation in the chemical composition of wheat flour among studies can be ascribed to differences in genotypic characteristics, agronomic practices such as irrigation management, post-harvest processing including milling methods, and inherent varietal physiological and biochemical traits (22).

**Table 3:** Proximate composition and gluten content of newly developed wheat genotypes grown in the Western Himalayas.

**Variety**	**Moisture (%)**	**Protein (%)**	**Fat (%)**	**Ash (%)**	**Fiber (%)**	**Carbohydrate (%)**	**Energy (kcal/100 g)**	**Dry gluten (%)**	**Wet gluten (%)**
KWQ-21-1	13.10± 0.05^B^	11.39 ± 0.05^A^	0.57 ± 0.01^D^	0.15 ± 0.06^DE^	1.60 ± 0.11^C^	73.17 ± 0.04^H^	343.19 ± 0.01^G^	13.20 ± 0.05^A^	33.87 ± 0.04^A^
KWQ-21-2	12.56± 0.11^C^	10.20 ± 0.06^B^	0.13 ± 0.02^G^	0.29 ± 0.02^BC^	1.43± 0.08^CDE^	75.37 ± 0.04^D^	343.16 ± 0.05^G^	9.82 ± 0.09^FG^	27.50 ± 0.08^E^
KWQ-21-3	12.19± 0.04^D^	9.55± 0.08^C^	0.66 ± 0.05^C^	0.31 ± 0.01^BC^	2.25± 0.11^B^	75.13 ± 0.05^E^	344.65 ± 0.07^D^	10.80 ± 0.08^D^	28.18 ± 0.05^D^
KWQ-21-4	11.37± 0.02^E^	10.40 ± 0.08^B^	0.77 ± 0.04^B^	0.32 ± 0.01^B^	3.32 ± 0.14^A^	73.81 ± 0.05^G^	343.76 ± 0.05^E^	11.18 ± 0.07^C^	31.43 ± 0.03^B^
SKW 374	11.40± 0.05^E^	9.30± 0.05^D^	0.46 ± 0.06^E^	0.42 ± 0.01^A^	2.14 ± 0.03^B^	76.27 ± 0.05^B^	346.42 ± 0.08^B^	9.30 ± 0.08^G^	25.16 ± 0.09^H^
KWQ-21-6	13.54± 0.03^A^	8.73± 0.08^E^	0.35 ± 0.03^F^	0.13 ± 0.02^E^	2.41 ± 0.09^B^	74.83 ± 0.05^F^	337.47 ± 0.05^H^	10.15 ± 0.05^E^	27.23 ± 0.05^F^
KWQ-21-7	12.56± 0.08C	10.40± 0.05^B^	0.56 ± 0.02^D^	0.11 ± 0.02^E^	1.44 ± 0.12^CD^	74.85 ± 0.07^F^	346.13 ± 0.04^C^	11.77 ± 0.05^B^	29.74 ± 0.07^C^
Shalimar Wheat-2	12.28± 0.07^D^	9.20± 0.05^D^	0.15 ± 0.01^G^	0.46 ± 0.02^A^	1.17 ± 0.02^DE^	76.72 ± 0.05^A^	344.73 ± 0.10^D^	9.88 ± 0.06^F^	26.55 ± 0.02^G^
SKW 357	13.26± 0.04^B^	8.06± 0.01^F^	1.02 ± 0.07^A^	0.20 ± 0.01^D^	1.11 ± 0.01^E^	76.37 ± 0.04^B^	346.73 ± 0.09^A^	9.10 ± 0.02^G^	24.73 ± 0.04^I^
Shalimar Wheat-3	12.18± 0.02^D^	9.23± 0.05^D^	0.48 ± 0.01^E^	0.26 ± 0.01^C^	2.31 ± 0.08^B^	75.54 ± 0.05^C^	343.41 ± 0.03^F^	8.78 ± 0.05^H^	23.27 ± 0.05^J^

Data are presented as the mean ± SD. Means with different superscripts in columns differ significantly (*p* ≤ 0.05). *n* = 3.

#### 3.3.2 Dry and wet gluten content

The gluten content (wet and dry) of the flour obtained from selected wheat genotypes had significant (*p* ≤ 0.05) differences (Table 3). The wet gluten content of different wheat genotypes varied significantly (*p* ≤ 0.05) and was found in the range of 23.27% to 33.87%. KWQ-21-1 had the highest wet gluten content (33.87%), whereas Shalimar Wheat-3 (23.27%) had the lowest. The dry gluten of different wheat flours was noted to be from 8.78% to 13.20%. KWQ-21-1 exhibited the highest dry gluten, whereas Shalimar Wheat-3 had a lower dry gluten content. The concentration of gluten-forming proteins in wheat flour is commonly assessed through wet gluten content, which is a critical determinant of dough rheological behavior and baking performance. Genotypic variation and cultivar-specific traits significantly influence gluten levels (23), thereby accounting for the substantial differences in gluten content observed among the wheat varieties in this study. Rani et al. (8) reported the gluten content of six wheat cultivars cultivated under identical environmental conditions, reporting dry gluten levels ranging from 7.16% to 11.24% and wet gluten content from 20.60% to 41.24%, which are closely aligned with the studied results. Based on the dry and wet gluten contents, genotypes such as KWQ-21-1 and KWQ-21-4 can be classified as hard wheat genotypes suitable for strong flour applications, while genotypes such as Shalimar Wheat-3, SKW 357, and SKW 374 fall into the soft wheat category, suitable for weak flour applications. Simultaneously, on the gluten content, KWQ-21-1 and KWQ-21-4, with high dry and wet gluten percentages, are suitable for bread and pasta production; genotypes such as KWQ-21-2, KWQ-21-3, and KWQ-21-7, with moderate gluten levels, are appropriate for chappatis and noodles, while Shalimar Wheat-3, SKW 357, and SKW 374, having low gluten content, are better suited for biscuits and confectionery products.

#### 3.3.3 Color characteristics of flour

The color parameters (L*, a*, and b*) of the wheat flour from selected genotypes varied from 66.83 to 80.16, 2.87 to 3.54, and 10.44 to 15.03, respectively (Table 2). The L* parameter of wheat flour reflected a significant (*p* ≤ 0.05) difference from each other. SKW 357 had the highest value (80.16), and KWQ-21-2 had the lowest value (66.83). For a* parameter, SKW 357 flour reflected a significantly (*p* ≤ 0.05) higher value than other genotypes, indicating redness in the wheat flour sample. Shalimar Wheat-3 showed high yellowness compared to other genotypes' flour due to a significantly (*p* ≤ 0.05) higher value of b*. The flour of KWQ-21-1 showed the lowest value of b*. The Hue angle (H°) and Chroma (C^*^) values differed significantly (*p* ≤ 0.05) among the different flours and varied from 71.90 to 76.86° and 10.98 to 15.43, respectively. Wheat genotypes Shalimar Wheat-3 exhibited the highest value, and KWQ-21-1 exhibited the lowest value for Hue angle (H°). The maximum Chroma (C^*^) value was observed for Shalimar Wheat-3, and the minimum in KWQ-21-1. Punia et al. (24) reported color parameters for 10 Indian wheat varieties, with L^*^ values ranging from 71.2 to 79.4, a^*^ values from 1.55 to 2.45, and b^*^ values from 8.49 to 12.00. These findings are closely aligned with the results observed in this study. Differences in flour color among wheat genotypes are predominantly attributed to variations in ash content and the extent of bran contamination during milling, with minor contributions from intrinsic pigments such as flavonoids, carotenoids, anthocyanins, and specific phenolic compounds (10).

### 3.4 Flour performance properties

#### 3.4.1 Solvent retention capacity

The water solvent retention capacity (WSRC), sodium carbonate solvent retention capacity (SCSRC), lactic acid solvent retention capacity (LASRC), and sucrose solvent retention capacity (SUSRC) were reported, ranging from 57.14% to 67.13%, 63.40% to 73.52%, 72.73% to 102.13%, and 72.42% to 87.44%, respectively, and varied significantly (*p* ≤ 0.05) for different wheat flours (Table 4). WSRC was noted as maximum for KWQ-21-3 and minimum for SKW 357. SCSRC values were highest for KWQ-21-6 and lowest for SKW 357. LASRC was observed as maximum for KWQ-21-7 and minimum for KWQ-21-4. SUSRC values differed significantly, with the highest percentage in KWQ-21-6 (87.44%) and the lowest in Shalimar Wheat-3 (72.42%). SRC measures the swelling behavior of specific wheat flour components in response to component-specific solvents, such as LASRC, WSRC, SCSRC, and SUSRC, each of which interacts preferentially with different flour constituents. The solvent retention capacity of flour is primarily influenced by the composition and functionality of its constituent proteins, arabinoxylans (pentosans), glycoproteins, and the extent of starch damage (25). The WSRC is influenced by all the flour components (starch, gluten, arabinoxylan, and gliadin), SUSRC by pentosans, SCSRC by damaged starch, and LASRC by glutenin (8). SRC contributes to improved product quality by offering information on the various chemical components of flour during dough formation, as well as its rheological properties throughout baking and processing (11, 12), thereby supporting better end-product utilization. The values of WSRC (57.14% to 67.13%), SCSRC (63.40% to 73.52%), LASRC (72.73% to 102.13%), and SUSRC (72.42% to 87.44%) found in this study were comparable to those observed by Holkovicova et al. (11), who reported WSRC of 68.03% to 72.74%, LASRC of 114.71% to 124.15%, SCSRC of 73.62% to 89.85%, and SUSRC of 94.36% to 106.12%. Baljeet et al. (26) reported that the solvent retention capacities of wheat flour varied within the following ranges: 59.03%−80.73% for WSRC, 55.63%−112.30% for SCSRC, 80.66%−128.33% for LASRC, and 101.50%−119.43% for SUSRC. Higher SRC values generally indicate stronger functional characteristics of specific flour components, which are correlated with improved dough handling properties and superior baking quality (26, 27). The wheat varieties evaluated in this study exhibit medium-to-strong gluten strength and are classified within the soft-to-semi-soft texture range. Their relatively low LASRC values suggest a greater suitability for the production of soft-textured products such as cakes, chapattis, and biscuits. Sharma et al. (28) reported higher SRC values in certain hard wheat cultivars, attributing these to their higher protein content, greater gluten strength, increased levels of damaged starch (DS), enhanced water absorption capacity (WAC), and elevated arabinoxylan concentrations. The variations in the SRC values may result from differences in the genetic composition of the genotypes as well as in their milling properties, particularly regarding starch damage and extractable pentosan content (8).

**Table 4:** Solvent retention capacity (SRC) and alkaline water retention capacity (AWRC) of flours of the newly developed wheat genotypes grown in the Western Himalayas.

**Variety**	**Water (WSRC)**	**Sucrose (SUSRC)**	**Lactic acid (LASRC)**	**Sodium carbonate (SCSRC)**	**Gluten performance index (GPI)**	**Alkaline (AWRC)**
KWQ-21-1	63.03 ± 0.05^D^	80.62 ± 0.05^F^	82.23 ± 0.06^F^	71.15 ± 0.05^D^	0.54 ± 0.01^EF^	67.22 ± 0.05^D^
KWQ-21-2	64.21 ± 0.07^C^	82.08 ± 0.05^D^	97.50 ± 0.07^B^	72.24 ± 0.05^C^	0.63 ± 0.03^A^	67.50 ± 0.03^C^
KWQ-21-3	67.13 ± 0.05^A^	85.76 ± 0.04^B^	94.88 ± 0.05^C^	72.66 ± 0.06^B^	0.59 ± 0.07^ABCDE^	62.64 ± 0.05^H^
KWQ-21-4	62.53 ± 0.06^E^	81.31 ± 0.06^E^	72.73 ± 0.06^I^	70.54 ± 0.05^E^	0.45 ± 0.05^G^	63.55 ± 1.06^G^
SKW 374	57.49 ± 0.05I	79.32 ± 0.05^G^	94.28 ± 0.05^D^	71.22 ± 0.05^D^	0.62 ± 0.04^AB^	67.61 ± 0.04^B^
KWQ-21-6	65.34 ± 0.03^B^	87.44 ± 0.05^A^	97.64 ± 0.06^B^	73.52 ± 0.06^A^	0.60 ± 0.08^ABCD^	68.22 ± 0.06^A^
KWQ-21-7	62.20 ± 0.05^F^	85.54 ± 0.05^C^	102.13 ± 0.07^A^	72.69 ± 0.05^B^	0.62 ± 0.03^ABC^	66.46 ± 0.07^F^
Shalimar Wheat-2	61.85 ± 0.04^G^	74.90 ± 0.05^H^	84.21 ± 0.05^E^	69.33 ± 0.06^G^	0.56 ± 0.05^BDEF^	66.67 ± 0.06^E^
SKW 357	57.14 ± 0.05^J^	74.74 ± 0.6^H^	80.45 ± 0.04^G^	63.40 ± 0.10^H^	0.56 ± 0.02^BDEF^	66.53 ± 0.09^F^
Shalimar Wheat-3	59.55 ± 0.08^H^	72.42 ± 0.04^I^	74.13 ± 0.03^H^	69.80 ± 0.05^F^	0.52 ± 0.01^F^	61.23 ± 0.05^I^

Data are presented as the mean ± SD. Means with different superscripts in columns differ significantly (*p* ≤ 0.05). *n* = 3.

The Gluten Performance Index (GPI) serves as an indicator of flour functionality, providing a quantitative measure of gluten strength and its potential contribution to baking performance (12). The GPI of flour of different wheat genotypes varied significantly (*p* ≤ 0.05) and ranged from 0.45 to 0.63 (Table 4). KWQ-21-2 had the highest GPI (0.63), while KWQ-21-4 showed the lowest GPI (0.45). Duyvejonck et al. (29) reported that GPI is a better parameter in predicting the bread-making qualities of wheat cultivars. Lindgren and Simsek (30) reported that GPI is a better indicator of end-product quality in hard wheat varieties than any other SRC values. Soft wheat cultivars have generally low GPI values ranging from 0.52 to 0.69. The GPI values found in our study were comparable to those observed by Joe et al. (31), who reported the GPI in the range of 0.53–0.69. However, low values of GPI have been observed by Rani et al. (8) (0.48–0.55) for different wheat varieties. The Gluten Performance Index (GPI) is closely associated with LASRC and may be negatively affected by higher SCSRC and SUSRC values. In our study, the relatively uniform values of LASRC, SUSRC, and SCSRC may account for the observed GPI levels and reflect the characteristics of the wheat genotypes utilized.

#### 3.4.2 Alkaline water retention capacity

Alkaline water retention capacity (AWRC) of wheat flour obtained from selected genotypes is given in Table 4. The AWRC of different flours varied significantly (*p* ≤ 0.05) from 61.23% to 68.22%. The highest AWRC value (68.22%) was observed for KWQ-21-6, while the lowest value (61.23%) was noted for Shalimar Wheat-3. Alkaline water retention capacity (AWRC) testing is performed to assess the water absorption and retention properties of flour, which are indicative of its suitability for producing cookies with desirable spread and texture characteristics. Flours with low AWRC values are generally preferred for cookie production, as they are associated with minimal water absorption and limited gluten formation, resulting in cookies with larger diameters (32). The flour fraction, composed of damaged starch, proteins, pentosans, and glycoproteins, is believed to be accountable for retaining alkaline water. The AWRC values of 61.23% to 68.22% found in this study were comparable to those reported by Moiraghi et al. (33), who reported AWRC values of 51 soft wheat genotypes, ranging from 64.6% to 70.1%. The variation in AWRC observed among the evaluated wheat varieties may be attributed to the synergistic effects of higher damaged starch content and inherent genotypic differences.

### 3.5 Functional properties of flour

#### 3.5.1 Water absorption capacity

The water absorption capacity (WAC) of wheat flour obtained from different genotypes is given in Table 5. The WAC differed significantly (*p* ≤ 0.05) from 132.31% to 155.85%. The WAC was observed to be maximum for KWQ-21-6 (155.85%) and minimum for Shalimar Wheat-2 (132.31%). WAC is a critical parameter in bread baking, as higher water absorption contributes to improved dough handling properties and delays staling by retaining more moisture within the bread matrix (34). WAC is consistently associated with increased amylose leaching and solubility, alongside the disruption or loss of starch crystallinity (35). The WAC of the wheat flour found in this investigation (132.31% to 155.85%) was close to that reported in previous research. Earlier studies reported WAC values of 132% to 176% by Chandra et al. (35) and 147.67% to 179% by Narwal et al. (36) for different wheat flour samples. Increased water absorption by flour is often indicative of a higher concentration of hydrophilic components, particularly non-starch polysaccharides and proteins, which possess strong water-binding capacities. Proteins exhibit amphiphilic properties, containing both hydrophilic and hydrophobic regions, which enable them to interact effectively with water and other components in food systems. The observed variation among different flours may be attributed to differences in protein content, the extent of protein–water interactions, and the conformational properties of the protein molecules (37).

**Table 5:** Functional properties of the newly developed wheat genotypes grown in the Western Himalayas.

**Variety**	**Water absorption capacity (WAC) (%)**	**Oil absorption capacity (OAC) (%)**	**Emulsion capacity (%)**	**Emulsion stability (%)**	**Swelling capacity (ml)**	**SDS-sedimentation volume (ml)**	**Falling number (seconds)**
KWQ-21-1	138.44 ± 0.12^E^	139.53 ± 0.11^B^	32.58 ± 0.05^A^	25.75 ± 0.11^B^	15.00 ± 0.57^CDE^	51.00 ± 0.57^C^	480 ± 0.56^G^
KWQ-21-2	151.21 ± 0.05^B^	128.90 ± 0.01^C^	27.74 ± 0.03^C^	22.40 ± 0.08^C^	14.66 ± 0.88^CDE^	53.00 ± 0.58^C^	502 ± 0.88^F^
KWQ-21-3	145.56 ± 0.04^C^	118.89 ± 0.06^E^	28.70 ± 0.07^B^	21.15 ± 0.05^E^	17.00 ± 0.57^AB^	46.33 ± 0.88^D^	533 ± 0.87^E^
KWQ-21-4	151.77 ± 0.06^B^	117.86 ± 0.05^F^	26.82 ± 0.05^E^	22.22 ± 0.06^C^	16.00 ± 0.58^BC^	36.00 ± 0.57^F^	838 ± 0.57^A^
SKW 374	135.29 ± 0.06^F^	116.16 ± 0.02_G_	25.59 ± 0.08^G^	20.62 ± 0.05^F^	18.00 ± 0.57^A^	45.00 ± 0.57^D^	270 ± 0.60^I^
KWQ-21-6	155.85 ± 2.0^A^	114.70 ± 0.06^H^	24.67 ± 0.05^H^	18.87 ± 0.07^G^	16.00 ± 0.56^BCD^	55.66 ± 0.88^B^	503 ± 0.58^F^
KWQ-21-7	136.15 ± 0.04^F^	121.47 ± 0.10^D^	20.52 ± 0.08^I^	15.63 ± 0.05^H^	14.00 ± 0.60^CEF^	73.66 ± 0.88^A^	430 ± 0.62^H^
Shalimar Wheat-2	132.31 ± 0.02^G^	146.87 ± 0.06^A^	32.56 ± 0.08^A^	26.52 ± 0.05^A^	12.00 ± 0.57^F^	44.50 ± 0.57^DE^	660 ± 0.60^C^
SKW 357	138.38 ± 0.04^E^	108.65 ± 0.06^J^	26.48 ± 0.11^F^	18.71 ± 0.09^G^	12.50 ± 0.61^F^	42.50 ± 0.60^E^	642 ± 0.61^D^
Shalimar Wheat-3	143.23 ± 0.06^D^	113.20 ± 0.06^I^	27.34 ± 0.05^D^	21.72 ± 0.05^D^	16.00 ± 0.57^BCD^	38.00 ± 0.57^F^	707 ± 0.59^B^

Data are presented as the mean ± SD. Means with different superscripts in columns differ significantly (*p* ≤ 0.05). *n* = 3.

#### 3.5.2 Oil absorption capacity

The oil absorption capacity (OAC) of different wheat flours varied significantly (*p* ≤ 0.05) between 108.65% and 146.87% (Table 5). The highest OAC value (146.87%) was observed for Shalimar Wheat-2, while the lowest value (108.65%) was noted for SKW 357. OAC reflects the ability of flour to bind or interact with oil, indicating the presence and availability of non-polar side chains in proteins and other macromolecules. Intrinsic properties such as protein conformation, the specific amino acid profile, and the spatial arrangement of polar and hydrophobic residues significantly influence the oil absorption capacity of food proteins. A high OAC in wheat flour suggests its suitability for incorporation into lipid-rich formulations, such as baked goods, where oil retention contributes to texture, mouthfeel, and flavor stability (36). The OAC of the wheat flour found in this investigation (108.65% to 146.87%) was close to that reported in previous research. Earlier studies reported oil absorption capacity (OAC) values of 114%−142.67% (36) for different wheat flours. The observed variations in OAC among different wheat flours may be attributed to differences in the quantity and nature of hydrophobic proteins, which exhibit strong oil-binding capacity (38).

#### 3.5.3 SDS-sedimentation value

The sodium dodecyl sulfate-sedimentation value (SDS-SV) differed significantly (*p* ≤ 0.05) from 36 ml to 73.66 ml (Table 5) among different wheat genotypes. The SDS-SV was noted as maximum for KWQ-21-7 (73.66 ml) and minimum for KWQ-21-4 (36 ml). SDS value helps to forecast the gluten strength and baking quality of wheat flour, as well as providing information on the protein quantity of the flour. The swelling of glutenin strands is responsible for the SDS-sedimentation value; higher gluten strength dough shows more swelling in the SDS solution, which raises the sedimentation values. Wheat varieties with SDS-SV below 30 ml are generally suited for cookie production due to their weaker gluten strength; those with values between 35 and 45 ml are appropriate for chapatti or pasta making, while varieties exhibiting values above 45 ml are preferred for high-quality bread production, reflecting stronger gluten-forming potential (39). The SDS-SV found in our study was comparable to those reported by Siddiqi et al. (10) and Rani et al. (8), which were in the range of 34.50–49.50 ml and 47–72 ml. Kundu et al. (39) also reported the SDS-sedimentation values of 50 Indian wheat varieties in the range between 28 and 61.50 ml. Variations in SDS-SV may be attributed to genotypic differences among wheat varieties and the influence of irrigation management practices. Despite the observed variations, all wheat varieties were considered well-suited for chappatis and cake preparation, with specific genotypes such as KWQ-21-7 exhibiting quality traits indicative of suitability for bread-making applications.

#### 3.5.4 Emulsion capacity

The emulsion capacity (EC) of flour of different wheat genotypes varied significantly (*p* ≤ 0.05) and ranged from 20.52% to 32.58% (Table 5). KWQ-21-1 had the highest emulsion capacity (32.58%), while KWQ-21-7 showed the lowest emulsion capacity (20.52%). Flour's protein content plays a key role in its emulsifying properties due to the presence of both water-attracting (hydrophilic) and water-repelling (hydrophobic) amino acids. Hydrophobic interactions can occur between the non-polar side chains of amino acids and the hydrocarbon chains of lipids. The higher EC in the flours could be due to the proteins containing a high proportion of the polar and non-polar amino acids, and lower values are due to a low proportion of the polar and non-polar amino acids (40). Previously, Ocheme et al. (41) reported that the EC of wheat flours ranged from 27.58% to 37.04%, which is comparable to the findings of our studies. Variation in the emulsion capacity of flours could be due to differences in protein solubility and composition, which are influenced by genotype, climatic conditions, and agronomic practices. The emulsifying properties of proteins have been linked to their hydrophobicity. Solubility, pH, and concentration are among the numerous factors that influence these properties.

#### 3.5.5 Emulsion stability

The emulsion stability of wheat flour obtained from selected genotypes is given in Table 5. Emulsion stability of different flours varied significantly (*p* ≤ 0.05), ranging from 15.63% to 26.52%. The highest ES value (26.52%) was observed for Shalimar Wheat-2, while the lowest value (15.63%) was noted for KWQ-21-7. Emulsion stability (ES) refers to the ability of flour proteins to maintain a stable emulsion over time without phase separation. ES of flour varies due to differences in protein composition, solubility, and surface hydrophobicity, which are influenced by genotype, environmental conditions, and processing factors. Additional components such as starch and fiber may further modulate the ability of proteins to form stable interfacial films. The higher ES in the flours could be due to the proteins containing a high proportion of the polar and non-polar amino acids, and lower values are due to a low proportion of the polar and non-polar amino acids (40). Akoja et al. (42) reported the emulsion stability of wheat flour in the range between 5.22% and 8%. However, Arepally et al. (43) reported higher values of ES (29.16%−45.63%).

#### 3.5.6 Falling number

The falling number (FN) of flours of different wheat genotypes varied significantly (*p* ≤ 0.05), ranging from 270 to 838 seconds (Table 5). The FN was noted as maximum for KWQ-21-4 (838 seconds) and minimum for SKW 374 (270 seconds). The FN test evaluates the level of enzymatic activity, α-amylase, in a wheat flour or meal sample, with results typically reported in seconds. A high falling number indicates low endogenous α-amylase activity, reflecting minimal starch degradation and thus high starch integrity. This is generally associated with sound, non-sprouted grain and is considered a marker of superior flour quality for most baking applications, particularly where strong gel-forming properties and consistent dough performance are required. A low FN reflects elevated α-amylase activity, indicating significant starch hydrolysis typically resulting from pre-harvest sprouting. A low FN indicates excessive α-amylase activity, leading to increased hydrolysis of starch into simpler sugars, resulting in elevated sugar levels and reduced intact starch content. In contrast, a high falling number reflects low enzymatic activity, preserving native starch reserves and resulting in lower concentrations of degradation sugars. Variations in FN have been linked to differences in the extent of starch degradation, as well as the particle size and storage duration of flour. These factors can influence enzymatic activity and starch integrity, thereby affecting FN values (44). Previously, Kaur et al. (45) and Panghal et al. (46) reported FN values ranging from 320 to 967 seconds across 108 Indian wheat varieties. Similarly, Abraha et al. (47) observed FN values between 180 and 620 seconds in 27 Ethiopian wheat samples, which are comparable to the range observed in this study.

#### 3.5.7 Swelling capacity

The swelling capacity (SC) of wheat flour varied significantly (*p* ≤ 0.05), ranging from 12 to 18 ml (Table 5). SKW 374 had the highest swelling capacity (18 ml), while Shalimar Wheat-2 had the lowest (12 ml). Swelling capacity reflects the ability of starch granules to absorb and retain water upon heating, indicating the degree of granule hydration and gelatinization under thermal conditions (48). The SC of flour is impacted by factors such as variety, processing techniques, and particle size. An increased starch concentration in the flour contributes to greater SC, as a higher proportion of starch granules are available to absorb water and undergo gelatinization. Conversely, lower starch content is associated with reduced SC (49). The variations in swelling capacity of flour depend upon the extent of proteins, lipids, and amylose content present in the flour (50). Our results are consistent with those reported by Aniemema et al. (49), who observed swelling capacities of wheat flour ranging from 10.03 ml to 25.39 ml. Kumar et al. (48) also reported the swelling capacity of various flours to range between 17.16 and 31.33 ml.

### 3.6 SDS-PAGE of wheat flour

**Figure 1:**
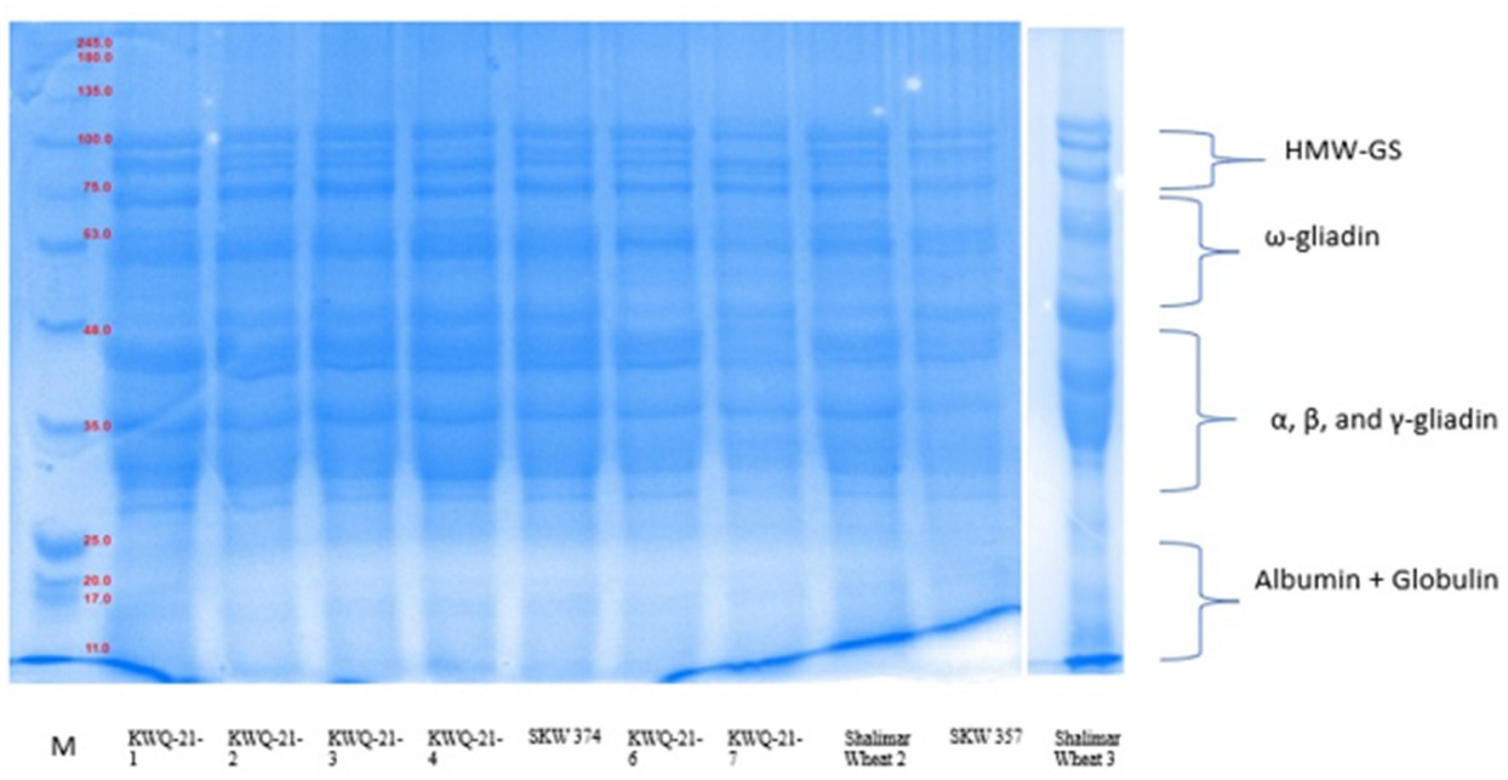
SDS-PAGE of total flour proteins under reducing conditions using 12% resolving gel. of newly developed wheat genotypes grown in Western Himalayas.

SDS-PAGE analysis of defatted wheat flour for protein examination of 10 wheat varieties under reduced conditions is given in Figure 1. The total number of polypeptide bands detected by SDS-PAGE across the wheat varieties ranged from 12 to 16, with molecular weights spanning from 11 to 111.5 kDa. Among high-molecular-weight glutenin subunits (HMW-GSs), proteins ranged between 76.5 and 111.5 kDa, ω-gliadin between 50.7 and 75 kDa, α-, β-, and γ-gliadin between 27.3 and 49.7 kDa, and albumins + globulins between 11 and 25.8 kDa. The total flour protein was reported by Siddiqi et al. (10), who found low-molecular-mass albumins in the molecular weight range of 4.4–26.9 kDa, α-, β-, and γ-gliadin/LMW-GS (27.1–50.1 kDa), ω-gliadin (Mw = 50.7–64.6 kDa), and HMW-GS in the molecular weight range of 65.1–120.8 kDa. The genetic composition of the varieties and the cultivation practices may be the cause of the differences in molecular mass in comparison to the two investigations. The five wheat genotypes (KWQ-21-1, KWQ-21-3, SKW374, KWQ-21-6, and Shalimar Wheat-2) exhibited four high-molecular-weight glutenin subunit (HMW-GS) bands, while five varieties (KWQ-21-1, KWQ-21-4, KWQ-21-7, SKW 357, and Shalimar Wheat-3) exhibited three bands of high-molecular-weight glutenin subunits (HMW-GSs). According to Anjum et al. (51), standard wheat has three to five HMW-GS. Depending on the variety, the number of polypeptides in the ω-gliadin varied from one to four. KWQ-21-6, Shalimar Wheat-2, SKW357, and Shalimar Wheat-3 resolved into three bands; KWQ-21-1 and KWQ-21-4 wheat types exhibited an appearance of two ω-gliadin polypeptides. KWQ-21-7 exhibited four ω-gliadin polypeptides, and KWQ-21-2, KWQ-21-3, and SKW374 exhibited one ω-gliadin polypeptide. High levels of heterogeneity were observed in α-, β-, and γ-gliadin/LMW areas, which correlate to the molecular mass range of 30.7–48 kDa, in terms of both quantity and intensity. To distinguish across wheat types, a genetic biomarker spanning 30.7 to 46.8 kDa can be employed. The protein bands, approximately 27.4 and 29.6 kDa, did not have sufficient clarity to allow for the identification of the various constituent subunits. The wheat types SKW357 and KWQ-21-3 were identified by the existence of distinct high-intensity bands at 43.7 and 44.5 kDa. The albumin and globulins (A + G) protein-corresponding polypeptides in the molecular mass range of 11–25.8 kDa exhibited nearly comparable molecular weights regardless of the wheat type. Similar findings in other studies have revealed little to no variability in either globulin or albumin (52).

The measured proportions of the various flour proteins are given in Table 6, Figure 1. High-molecular-weight glutenin subunits (HMW-GSs) constituted between 8.5% and 26.3% of the total extractable flour proteins. HMW-GS differed significantly (*p* ≤ 0.05) among the wheat genotypes. Among the wheat genotypes evaluated, SKW 374 exhibited the highest proportion of HMW-GS, while KWQ-21-1 showed the lowest. HMW-GS, though comprising only 5%−10% of total grain protein, accounts for up to 70% of the variation in bread-making quality, primarily due to its pivotal role in gluten polymer formation and dough viscoelastic properties. HMW-GS plays a central role in conferring dough elasticity, facilitating gas retention during fermentation, and thereby contributing to loaf volume and crumb structure (53). Extensive research has established a positive association between the relative abundance of HMW-GS and key indicators of bread-making quality, such as dough strength and loaf volume (54).

**Table 6:** Proportion of total proteins in the flours of the newly developed wheat genotypes grown in the Western Himalayas under reducing conditions.

**Variety**	**HMW-GS**	**ω-gliadin**	**α-, β-, and γ-gliadin/LMW-GS**	**A+G**	**HMW-GS/LMW-GS**
KWQ-21-1	8.50 ± 0.76^I^	19.60 ± 0.59^B^	50.10 ± 0.63^D^	21.70 ± 0.66^D^	0.16 ± 0.78^A^
KWQ-21-2	18.80 ± 0.68^D^	6.00 ± 0.72^G^	48.60 ± 0.68^F^	26.60 ± 0.63^A^	0.38 ± 0.63^A^
KWQ-21-3	16.10 ± 0.72^F^	4.50 ± 0.75^H^	58.40 ± 0.64^A^	21.00 ± 0.68^E^	0.27 ± 0.70^A^
KWQ-21-4	17.30 ± 0.70^E^	7.00 ± 0.70^F^	49.70 ± 0.65^DE^	25.90 ± 0.56^B^	0.34 ± 0.66^A^
SKW 374	26.30 ± 0.62^A^	5.50 ± 0.73^G^	49.20 ± 0.66^E^	19.00 ± 0.69^F^	0.53 ± 0.58^A^
KWQ-21-6	15.40 ± 0.74^GH^	24.50 ± 0.68^A^	42.50 ± 0.78^H^	17.50 ± 0.70^G^	0.36 ± 0.65^A^
KWQ-21-7	20.10 ± 0.65^C^	14.60 ± 0.67^D^	42.60 ± 0.70^H^	22.70 ± 0.60^C^	0.47 ± 0.56^A^
Shalimar Wheat-2	24.60 ± 0.61^B^	10.20 ± 0.69^E^	54.30 ± 0.60^C^	11.60 ± 0.70^H^	0.45 ± 0.60^A^
SKW 357	15.60 ± 0.73^FG^	4.80 ± 0.74^H^	57.20 ± 0.56^B^	22.40 ± 0.63^C^	0.27 ± 0.69^A^
Shalimar Wheat-3	14.90 ± 0.75^H^	18.30 ± 0.64^C^	44.90 ± 0.69^G^	21.80 ± 0.65^D^	0.33 ± 0.68^A^

Data are presented as the mean ± SD. Means with different superscripts in columns differ significantly (*p* ≤ 0.05). *n* = 2. HMW-GS, high-molecular-weight glutenin subunit; LMW-GS, low-molecular-weight glutenin subunit.

The ω-gliadin fraction exhibited significant (*p* ≤ 0.05) variation among wheat genotypes, with its proportion of total extractable wheat proteins ranging from 4.5% in KWQ-21-3 to 24.5% in KWQ-21-6. Among the α-, β-, and γ-gliadin/LMW-GS fractions, the α-gliadin/LMW-GS fraction was the most abundant and exhibited significant variation (*p* ≤ 0.05), ranging from 42.5% in KWQ-21-6 to 58.4% in KWQ-21-3. The findings revealed that low-molecular-weight gliadins were present at higher concentrations than ω-gliadins. Distinct gliadin subfractions have been shown to influence dough properties differently, depending on their specific biochemical characteristics. The combined proportion of A and G fractions varied significantly (*p* ≤ 0.05), ranging from 11.6% to 26.6% of the total flour proteins as determined by densitometric analysis of SDS-PAGE gels. SDS-PAGE analysis of wheat flour proteins revealed significant variation in the relative abundance of different protein fractions, particularly A + G and low-molecular-weight glutenin subunits (LMW-GSs). The proportions of total flour proteins obtained in our study are comparable to those observed by Rani et al. (8), who reported total flour proteins in the range of HWM-GS (13.57% to 18.75%), ω-gliadin (5.73% to 15.08%), α-, β-, and γ-gliadin/LMW-GS (37.79% to 41.16%), and A + G (25.01% to 42.75%). The ratio of HMW-GS to LMW-GS varied significantly (*p* ≤ 0.05) between 0.16 and 0.53. Greater HMW-GS/LMW-GS ratios in wheat cultivars are typically linked to better rheological and bread-making characteristics. Variations in the proportions of different protein fractions may be attributed to genetic makeup and agronomic practices influencing protein expression.

### 3.7 FTIR spectral analysis of wheat flour

**Figure 2:**
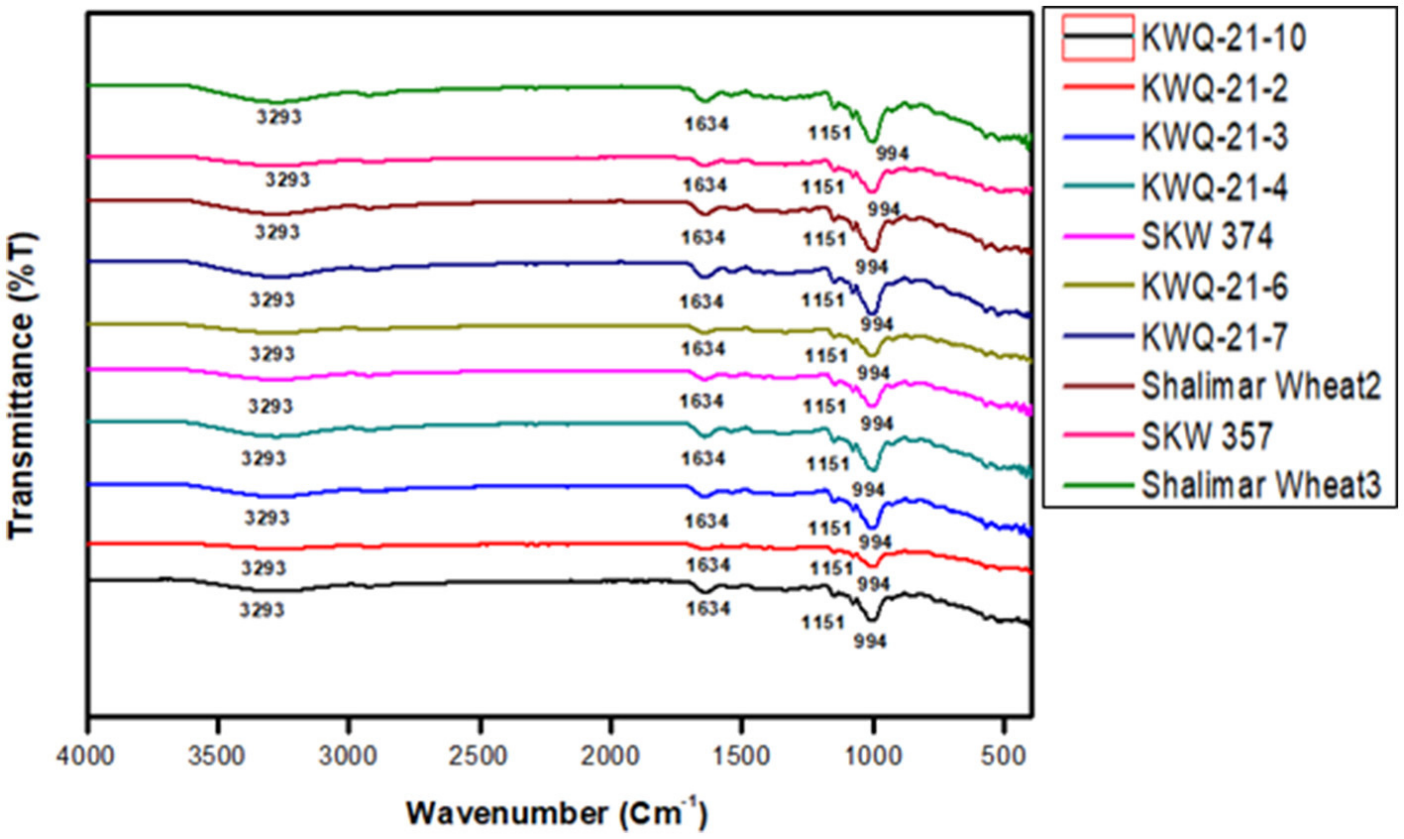
FTIR Spectra of newly developed wheat genotypes grown in Western Himalayas.

Fourier transform infrared (FTIR) spectroscopy is a rapid, non-destructive analytical method employed to characterize structural properties. Figure 2 presents the FTIR spectra of various wheat flour samples over the wavenumber range of 4,000 to 400 cm^−1^. Multiple absorption peaks were detected across distinct regions of the spectra. Characteristic absorption peaks were identified within the spectral regions of 900–1,000 cm^−1^, 1,500–1,650 cm^−1^, and 3,000–3,500 cm^−1^ for all the wheat genotypes. Only slight variations were observed in the FTIR spectra among the wheat flour samples. The first absorption peak was observed at 994 cm^−1^, which is attributed to C–O stretching vibrations or asymmetric stretching of C–OH groups, corresponding to polysaccharide structures such as starch. These observations are consistent with the findings of Iqbal et al. (55). The absorption band at 1,634 cm^−1^ is likely associated with C=O stretching vibrations, corresponding to the amide I band, which arises primarily from the stretching of the carbonyl (C=O) group in peptide linkages, indicative of protein secondary structure. These findings are consistent with the observations of Ahmad et al. (56). The absorption peak at 3,290 cm^−1^ is attributed to O–H stretching linked to hydrogen-bonded hydroxyl groups (57). The consistent peak profiles across all genotypes suggest a uniformity in their chemical composition. At 994 cm^−1^, a wavenumber typically attributed to C–O stretching vibrations in starch and polysaccharides, the transmittance values ranged from 81.99%T in Shalimar Wheat-3 to 92.95%T in KWQ-21-2. These differences suggest varying amounts of starch-related compounds across the genotypes, with KWQ-21-2 showing higher transmittance, possibly due to lower starch content or more ordered structures allowing greater transmission of IR light. The 1,634 cm^−1^ band, associated with the amide I region of proteins (C=O stretching vibrations of peptide bonds), displayed relatively high transmittance values across all genotypes, ranging from 93.05%T to 96.73%T. The highest value in KWQ-21-6 suggests a possible lower protein concentration or structural organization favoring higher transmittance. In contrast, KWQ-21-7 and Shalimar Wheat-3 exhibited slightly lower transmittance, potentially reflecting higher protein content or less ordered protein structures. For the 3,290 cm^−1^ region, representing O–H stretching vibrations and linked to hydrogen-bonded hydroxyl groups (indicative of water content or hydration-related structures), transmittance varied from 92.04%T in Shalimar Wheat-3 to 97.24%T in KWQ-21-2. The higher transmittance in KWQ*-*21-2 might indicate reduced water content or more crystalline structures, while the lower transmittance in Shalimar Wheat-3 suggests greater hydrogen bonding or moisture presence. Variations in FTIR transmittance are likely attributable to different wheat genotypes with differences in the content of proteins, lipids, including fatty acid profiles, and polysaccharides (58).

### 3.8 Pearson's correlation coefficient

Wheat quality can be described by a combination of various factors such as protein content, dry, and wet gluten, solvent retention capacity (SRC), sedimentation value, oil absorption capacity, water absorption capacity, emulsion capacity, emulsion stability, albumin and globulin content, gliadin subunit proportions, percentage of glutenin subunits, and Gli/Glu ratio.

Pearson's correlation coefficients between the wheat grains and different flour constituents are presented in Table 7. Protein content (PC) of flour exhibited a strong, significant correlation with wet gluten content (*r* = 0.839*, p* ≤ 0.01) and dry gluten (*r* = 0.824*, p* ≤ 0.01), which is expected since gluten proteins are the primary contributors to the total protein in flour. This indicates that as the protein content of flour increases, both wet and dry gluten contents also increase proportionally and significantly. These strong correlations emphasize the biochemical link between protein content and gluten formation. This relationship is important for evaluating wheat quality, especially in applications where strong gluten is needed. The high correlation coefficients and significance levels also suggest that protein content can be used as a predictive marker for gluten strength and quantity in flour quality assessment programs (59). A positive correlation between protein content (PC) and gluten (both dry and wet) has also been reported earlier by Siddiqi et al. (10).

**Table 7:** Correlation between wheat grain characteristics and various flour parameters.

	**PC**	**DG**	**WG**	**AWC**	**WSRC**	**SUSRC**	**LASRC**	**SCSRC**	**GPI**	**WAC**	**OAC**	**EC**	**ES**	**SC**	**SDS**	**FN**	**HMW-GS**	**ω-gliadin**	**α-, β-, and γ-gliadin **	**AG**	**HMW/LMW-GS**	**ACF**	L^*^g	a^*^g	b^*^g	L^*^f	a^*^f	b^*^f
PC	1																											
DG	**0.824** ^**^	1																										
WG	**0.839** ^**^	**0.962** ^**^	1																									
AWC	0.002	0.146	0.162	1																								
WSRC	0.371	0.476	0.494	−0.066	1																							
SUSRC	0.317	0.512	0.510	0.274	**0.735** ^*^	1																						
LASRC	0.023	0.104	0.018	0.524	0.393	0.712^*^	1																					
SCSRC	0.506	0.401	0.379	0.066	0.708^*^	0.744^*^	0.583	1																				
GPI	−0.161	−0.145	−0.255	0.562	0.123	0.397	0.915^**^	0.332	1																			
WAC	0.010	−0.026	0.125	−0.124	0.563	0.491	0.051	0.389	−0.143	1																		
OAC	0.507	0.447	0.444	0.302	0.281	−0.078	0.017	0.190	0.019	−0.361	1																	
EC	0.187	0.143	0.188	−0.068	0.135	−0.409	−0.460	−0.186	−0.334	−0.185	**0.683** ^*^	1																
ES	0.316	0.152	0.232	−0.050	0.120	−0.403	−0.469	−0.046	−0.381	−0.147	**0.751** ^*^	**0.951** ^**^	1															
SC	0.156	0.002	0.018	−0.268	0.177	0.372	0.145	0.554	0.038	0.379	−0.422	−0.206	−0.134	1														
SDS	0.288	0.433	0.297	0.490	0.303	0.621	**0.795** ^**^	0.476	0.662^*^	−0.096	0.136	−0.526	−0.517	−0.166	1													
FN	−0.102	−0.115	0.007	−0.588	0.025	−0.401	−0.775^**^	−0.401	−0.835^**^	0.280	−0.032	0.267	0.276	−0.333	−0.578	1												
HMW-GS	−0.300	−0.469	−0.438	0.215	−0.307	−0.111	0.317	0.043	0.365	−0.363	0.075	−0.227	−0.091	−0.014	0.038	−0.268	1											
ω-gliadin	0.147	0.288	0.180	0.128	0.214	0.167	0.021	0.354	−0.052	0.189	0.101	−0.057	−0.023	0.022	0.331	−0.046	−0.468	1										
α-, β-, and γ-gliadin	−0.233	−0.095	−0.058	−0.169	−0.037	−0.277	−0.252	−0.525	−0.136	−0.282	0.143	0.556	0.368	−0.224	−0.488	0.205	0.023	**−0.696** ^*^	1									
A+G	0.399	0.185	0.280	−0.241	0.048	0.191	−0.083	0.023	−0.164	0.448	−0.409	−0.331	−0.306	0.236	0.027	0.135	−0.393	−0.240	−0.131	1								
HMW/LMW-GS	−0.217	−0.405	−0.401	0.259	−0.278	0.025	0.426	0.225	0.426	−0.249	−0.027	−0.473	−0.289	0.066	0.256	−0.350	**0.928** ^**^	−0.198	−0.341	−0.303	1							
ACF	−0.173	−0.435	−0.332	−0.152	−0.198	−0.415	−0.206	−0.107	−0.098	−0.271	0.284	0.435	0.532	0.096	−0.581	0.106	**0.689** ^*^	−0.599	0.460	−0.370	0.439	1						
L^*^g	−0.432	−0.587	−0.587	0.026	−0.549	−0.381	−0.074	−0.152	0.001	−0.177	−0.220	−0.451	−0.289	−0.160	0.000	0.108	0.606	0.128	−0.493	−0.279	**0.745** ^*^	0.207	1					
a^*^g	**−0.767** ^**^	**−0.715** ^*^	**−0.695** ^*^	−0.381	−0.296	−0.302	−0.221	−0.273	−0.161	−0.026	−0.462	−0.114	−0.136	0.205	−0.541	0.250	0.483	−0.281	0.257	−0.419	0.364	0.533	0.469	1				
b^*^g	−0.528	**−0.701** ^*^	**−0.704** ^*^	−0.338	**−0.672** ^*^	**−0.746** ^*^	−0.430	−0.481	−0.249	−0.420	−0.154	−0.049	0.026	−0.195	−0.450	0.338	0.582	−0.225	0.055	−0.357	0.516	0.558	**0.789** ^**^	**0.705** ^*^	1			
L^*^f	−0.533	−0.147	−0.362	−0.175	−0.069	−0.015	0.127	−0.208	0.184	−0.246	−0.354	−0.257	−0.464	−0.147	0.224	−0.077	−0.188	0.331	0.040	−0.387	−0.139	−0.411	0.083	0.326	0.134	1		
a^*^f	−0.478	−0.100	−0.286	−0.295	−0.039	−0.042	−0.056	−0.241	0.005	−0.102	−0.433	−0.118	−0.349	0.018	0.014	0.011	−0.422	0.351	0.118	−0.257	−0.395	−0.441	−0.110	0.309	0.023	**0.941** ^**^	1	
b^*^f	−0.626	**−0.729** ^*^	**−0.813** ^**^	−0.464	−0.094	−0.314	−0.005	−0.085	0.131	0.071	−0.362	−0.230	−0.260	−0.040	−0.114	0.216	0.195	0.117	−0.150	−0.225	0.254	0.094	0.524	0.564	0.577	0.506	0.416	1

^**^Correlation is significant at the 0.01 level (2-tailed). ^*^Correlation is significant at the 0.05 level (2-tailed). PC, protein content; DG, dry gluten; WG, wet gluten; AWC, alkaline water retention capacity; WSRC, water solvent retention capacity; SUSRC, sucrose solvent retention capacity; LASRC, lactic acid solvent retention capacity; SCSRC, sodium carbonate solvent retention capacity; GPI, Gluten Performance Index; WAC, water absorption capacity; OAC, oil absorption capacity; EC, emulsion capacity; ES, emulsion stability; SC, swelling capacity; SDS, sedimentation volume; FN, falling number; HMW-GS, high-molecular-weight glutenin subunit; ω-gliadin, omega gliadin; α-, β-, and γ gliadin, alpha-, beta-, and gamma-gliadin; A+G, albumin and globulin; HMW/LMW-GS, ratio of high molecular weight to low molecular weight; ACF, ash content of flour; L^*^g, L-value of grain; a^*^g, a-value of grain; b^*^g, b-value of grain; L^*^f, L-value of flour; a^*^f, a-value of flour; and b^*^f, b-value of flour. Bold values represent significant correlations.

Dry gluten exhibited a strong positive correlation with wet gluten (r = 0.962, *p* ≤ 0.01). This indicates that as the content of dry gluten increases, the content of wet gluten also tends to increase proportionally. Dry gluten is essentially the solid component of wet gluten; their quantities are inherently linked. The strength of this correlation underscores their use in evaluating wheat quality, particularly for baking applications. Gulia and Khatkar (60) also reported a strong positive correlation (*r* = 0.92, *p* ≤ 0.01) between wet and dry gluten.

WSRC exhibited a positive relation with SCSRC (*r* = 0.708, *p* ≤ 0.05) and SUSRC (*r* = *0.735, p* ≤ 0.05), suggesting that the main factors influencing flour's ability to absorb water are starch degradation and pentosan concentration. The observed positive correlation between water and other SRC values results from water's ability to hydrate and enlarge the primary polymeric substances in flour. Similar results were reported by Holkovicova et al. (11). There was a positive correlation between SUSRC and LASRC (*r* = 0.712, *p* ≤ 0.05). SUSRC was positively correlated with SCSRC (*r* = 0.744, *p* ≤ 0.05). LASRC showed a highly significant positive correlation with GPI (r = 0.915, *p* ≤ 0.01). A strong, significant positive correlation was found between LASRC and SV (SV: *r* = 0.795, *p* ≤ 0.01). The positive relationship between LASRC, GPI, and SV can be attributed to their role as indicators of protein quality and gluten strength, which rely on the capacity of glutenin strands to expand in a lactic acid medium, thus implying that stronger gluten networks are reflected in both gluten performance and sedimentation values. A positive correlation between LASRC and SV was also reported by Karaduman et al. (61). LASRC showed a strong, significant negative correlation with falling number (*r* = −0.775*, p* ≤ 0.01), supporting its role as a marker for starch degradation. Labuschagne et al. (62) also showed similar results. GPI showed a highly significant negative correlation with falling (*r* = −0.835*, p* ≤ 0.01), reinforcing the antagonistic effect of enzymatic activity on gluten strength. Similar results were reported by Wrigley et al. (63).

High-molecular-weight glutenin subunits (HMW-GSs) showed a highly significant positive correlation with the ratio of HMW-GS to LMW-GS (*r* = 0.928, *p* ≤ 0.01). A positive significant correlation (*r* = 0.915, *p* ≤ 0.01) has also been reported by Siddiqi et al. (10). This underscores the pivotal role of HMW-GS in determining the glutenin composition of wheat flour. High-molecular-weight glutenin subunits (HMW-GSs) are integral to the formation of the gluten network, contributing significantly to dough elasticity and strength. A higher HMW-GS/LMW-GS ratio indicates a greater proportion of these subunits, which is associated with enhanced dough rheological properties and superior bread-making quality (53). This relationship highlights the importance of HMW-GS in flour functionality. HMW-GS also showed a significant positive correlation with ash content of flour (*r* = 0.689, *p* ≤ 0.05). ω-Gliadin showed significant negative correlation with α-, β-, and γ-gliadin (*r* = −0.696, *p* ≤ 0.05).

Oil absorption capacity showed a significant positive relation with emulsion capacity (*r* = 0.683*, p* ≤ 0.05) and emulsion stability (r = 0.751, *p* ≤ 0.05). This can be attributed to the functional role of hydrophobic proteins and other non-polar constituents in the sample matrix. These components not only enhance the ability of the flour to bind oil but also contribute to the formation and stabilization of oil-in-water emulsions. Higher OAC reflects a greater affinity for oil retention, which supports the development of a stable interfacial layer around dispersed oil droplets, thereby improving both the emulsion capacity and the stability of the emulsion system (38). A positive correlation was found between OAC and EC and ES, as reported by Punia et al. (25).

Emulsion capacity exhibited a very strong, significant positive correlation with emulsion stability (*r* = 0.951) with a significance level at 0.01%. The strong relationship highlights the functional role of flour constituents, particularly surface-active proteins such as glutenins and gliadins, in stabilizing oil–water interfaces. The significance of this relationship lies in its relevance to food processing and product quality (40). Therefore, emulsion capacity and stability can serve as valuable indicators of wheat flour's functional performance in emulsified food systems.

The flour color parameter b^*^ (yellowness) showed strong negative correlations with wet gluten (r = −0.813, *p* ≤ 0.01) and dry gluten (*r* = −0.729, *p* ≤ 0.05), indicating that as gluten concentration increases, the perceived yellowness of flour decreases. This may be due to dilution or entrapment of yellow pigments by protein matrices. Similar findings were reported by Wen et al. (64), who demonstrated inverse relationships between gluten content and flour color intensity. The b^*^ and a^*^ values of flour were highly correlated (r = 0.941, *p* ≤ 0.01), reflecting their combined influence on flour Hue and appearance. Additionally, the grain a^*^ value was negatively correlated with protein (r = −0.767, *p* ≤ 0.01), dry gluten (r = −0.715, *p* ≤ 0.05), and wet gluten (r = −0.695, *p* ≤ 0.05).

The L^*^ value of grains showed a positive correlation with b^*^ *(r* = 0.739, *p* ≤ 0.01). This suggests that as the grains become lighter (higher L^*^), they also tend to exhibit more yellowness (higher b^*^). The significant positive correlation between L^*^ and b^*^ implies that lighter-colored grains are more likely to be yellowish in tone. This is relevant in assessing visual grain quality, where brightness and yellowness are desirable traits, especially for consumer preference and processing quality in durum or bread wheat. The non-significant correlation with a^*^ suggests that red-green coloration is less consistently related to lightness and may vary due to genetic or environmental factors (8). There was a significant negative correlation between L^*^ and a^*^, b^*^ values as reported by Katyal et al. (19).

## 4 Conclusion

This study revealed significant variability in grain, flour, and functional properties among newly developed wheat genotypes grown in the Western Himalayas. The genotypes showed moderate protein and gluten content, weak-to-moderate gluten strength, and diverse solvent retention and SDS-sedimentation values, indicating their suitability for soft end-use products such as cakes, cookies, Kashmiri flat breads (Lavasa and Girda), and chappatis. Electrophoretic protein profiling highlighted notable genetic diversity, especially in gliadin and glutenin subunits. It provides a scientific basis for the targeted selection of genotypes for specific end-use applications. Furthermore, their adaptability to local growing seasons and rich nutritional profiles support rice–wheat crop rotation, which, otherwise, was impossible in this region. These findings provide a scientific foundation for the targeted breeding and selection of wheat genotypes tailored to specific functional applications and local agro-climatic conditions.

## Data Availability

The raw data supporting the conclusions of this article will be made available by the authors, without undue reservation.
