# Supplementary material for: Variability in grain, flour, functional properties and protein profiling in newly developed wheat genotypes grown in temperate climate of Western Himalayas
Source: Front Nutr. 2025 Jul 15;12:1604775. doi: 10.3389/fnut.2025.1604775 (PMC12304001; doi:10.3389/fnut.2025.1604775)
Supplement: Supplementary file 1 [file Table_1.docx]

***Supplementary Data:***

**Variability in grain, flour, functional properties and protein profiling in newly developed wheat genotypes grown in temperate climate of Western Himalayas**

*Taha Mukhtar****^1^****, Abida Jabeen****^1*^****, Syed Zameer Hussain****^1^****, Raashid Ahmad Siddiqi****^2^****, Shabir H. Wani****^3^****, Anju Mahendru-Singh^4^*, *Tawheed Amin****^1^****, Quraazah A. Amin****^1^,*** *Mohmad Sayeed Bhat****^1^***

**Table S1** Environmental conditions for the wheat growing seasons for 2018-2019 and 2019-2020 Mountain Research Centre for Field Crops (MRCFC) Khudwani (Anantnag-South Kashmir)

| **2018-2019** | | | | | | **2019-2020** | | | | | |  |
| --- | --- | --- | --- | --- | --- | --- | --- | --- | --- | --- | --- | --- |
| **Weeks** | **Period** | **Maximum air Temperature ( ^0^C)** | **Minimum air Temperature (^0^C)** | **Rainfall (mm)** | **Number of Sunshine hours** | **Weeks** | **Period** | **Maximum air Temperature ( ^0^C)** | **Minimum air Temperature (^0^C)** | **Rainfall (mm)** | **Number of Sunshine hours** |  |
| 38 | Sep. 17 – Sep. 23 | **28.28** | **9.5** | **1** | **8.9** | 35 | Aug.26 -Sep.01 | **26.6** | **13.6** | **1.8** | **20.6** |  |
|  |  |  |  |  |  |  |  |  |  |  |  |  |
| 39 | Sep. 24 – Sep. 30 | **26.1** | **8.93** | **0** | **6.63** | 36 | Sep.02 -Sep.08 | **28.5** | **14.8** | **0.2** | **20.3** |  |
|  |  |  |  |  |  |  |  |  |  |  |  |  |
| 40 | Oct. 01 – Oct. 07 | **24.29** | **5.2** | **0.3** | **6.87** | 37 | Sep.09 - Sep. 15 | **25.4** | **13.6** | **4.8** | **17.8** |  |
|  |  |  |  |  |  |  |  |  |  |  |  |  |
| 41 | Oct. 08 – Oct. 14 | **20.64** | **4.38** | **10** | **5.09** | 38 | Sep.16 –Sep.22 | **27** | **14.2** | **0.7** | **18** |  |
|  |  |  |  |  |  |  |  |  |  |  |  |  |
| 42 | Oct. 15 – Oct. 21 | **19.35** | **2.42** | **0.2** | **6.07** | 39 | Sep. 23 – Sep. 29 | **26.8** | **13** | **0.3** | **17.8** |  |
|  |  |  |  |  |  |  |  |  |  |  |  |  |
| 43 | Oct. 22 – Oct. 28 | **22.07** | **-0.36** | **0** | **7.23** | 40 | Sep. 30 – Oct.06 | **24.4** | **11.6** | **2.6** | **16.3** |  |
|  |  |  |  |  |  |  |  |  |  |  |  |  |
| 44 | Oct. 29 – Nov. 04 | **12.14** | **1.64** | **129.6** | **0.81** | 41 | Oct.07 – Oct.13 | **21.3** | **7.5** | **2.6** | **15.9** |  |
|  |  |  |  |  |  |  |  |  |  |  |  |  |
| 45 | Nov. 05 – Nov. 11 | **13.07** | **-1.1** | **0** | **1.73** | 42 | Oct.14 – Oct.20 | **18.7** | **7.1** | **5.5** | **13.4** |  |
|  |  |  |  |  |  |  |  |  |  |  |  |  |
| 46 | Nov. 12 – Nov. 18 | **11.5** | **1.64** | **8.6** | **1.77** | 43 | Oct.21 – Oct.27 | **12** | **3.6** | **10.5** | **10.6** |  |
|  |  |  |  |  |  |  |  |  |  |  |  |  |
| 47 | Nov. 19 – Nov. 25 | **13.64** | **-1.1** | **0** | **1.04** | 44 | Oct.28 –Nov.03 | **15.3** | **1.8** | **0.1** | **13.3** |  |
|  |  |  |  |  |  |  |  |  |  |  |  |  |
| 48 | Nov. 26 – Dec. 02 | **12.93** | **-2.5** | **0** | **0** | 45 | Nov. 04- Nov.10 | **13.3** | **1.9** | **0.2** | **11.4** |  |
|  |  |  |  |  |  |  |  |  |  |  |  |  |
| 49 | Dec.03 – Dec.09 | **14.33** | **0.18** | **138.4** | **2.66** | 46 | Nov. 11 – Nov. 17 | **13.8** | **-0.5** | **0.1** | **12.3** |  |
|  |  |  |  |  |  |  |  |  |  |  |  |  |
| 50 | Dec.10 – Dec.16 | **13.23** | **-0.14** | **276.6** | **2.17** | 47 | Nov. 18 – Nov. 24 | **13.5** | **-1** | **0.1** | **11.8** |  |
|  |  |  |  |  |  |  |  |  |  |  |  |  |
| 51 | Dec.17 – Dec.23 | 8.86 | **-0.11** | **553.2** | **1.45** | 48 | Nov. 25 -Dec.01 | 12 | **-1.2** | **0** | **10.8** |  |
|  |  |  |  |  |  |  |  |  |  |  |  |  |
| 52 | Dec.24 – Dec.31 | **12.78** | **-7.62** | **0** | **2.52** | 49 | Dec.02 -Dec.08 | **10.2** | **-1.1** | **1.3** | **9.7** |  |
|  |  |  |  |  |  |  |  |  |  |  |  |  |
| 1 | Jan.01 – Jan.07 | **12.96** | **-1.37** | **976.8** | **1.66** | 50 | Dec. 09 -Dec.15 | **7.9** | **-3.7** | **0** | **9.7** |  |
|  |  |  |  |  |  |  |  |  |  |  |  |  |
| 2 | Jan.08 – Jan.14 | **12.86** | **-1.8** | **1945** | **1.64** | 51 | Dec.16 - Dec. 22 | **8.2** | **-4.2** | **0.1** | **10** |  |
|  |  |  |  |  |  |  |  |  |  |  |  |  |
| 3 | Jan.15 – Jan.21 | **4.08** | **-1.91** | **3890** | **1.73** | 52 | Dec. 23 -Dec.29 | **5.9** | **-2.2** | **0.4** | **8.1** |  |
|  |  |  |  |  |  |  |  |  |  |  |  |  |
| 4 | Jan.22 – Jan.28 | **5.37** | **-2.5** | **33** | **1.03** | 1 | Dec. 30 -Jan.05 | **3.4** | **-1.5** | **4.8** | **5.9** |  |
|  |  |  |  |  |  |  |  |  |  |  |  |  |
| 5 | Jan.29 – Feb.04 | **6.57** | **-2.94** | **32** | **2.15** | 2 | Jan.06 – Jan.12 | **4.7** | **-3.7** | **0.7** | **8.7** |  |
|  |  |  |  |  |  |  |  |  |  |  |  |  |
| 6 | Feb.05 – Feb.11 | **5.96** | **-2.85** | **95.2** | **0.78** | 3 | Jan.13 – Jan.19 | **4.5** | **-2.2** | **0** | **7.8** |  |
|  |  |  |  |  |  |  |  |  |  |  |  |  |
| 7 | Feb.12 – Feb.18 | **8.86** | **-1.21** | **17.8** | **1.68** | 4 | Jan. 20 – Jan. 26 | **5.2** | **-1.9** | **1.8** | **8.7** |  |
|  |  |  |  |  |  |  |  |  |  |  |  |  |
| 8 | Feb.19 – Feb.25 | **7.71** | **0.05** | **19.5** | **2.88** | 5 | Jan.27– Feb.02 | **7.7** | **-3.1** | **0.3** | **11.3** |  |
|  |  |  |  |  |  |  |  |  |  |  |  |  |
| 9 | Feb.26 – Mar.04 | **12.93** | **-1.8** | **16.2** | **2.22** | 6 | Feb. 03 -Feb.09 | **8.4** | **-2.1** | **0.2** | **11.8** |  |
|  |  |  |  |  |  |  |  |  |  |  |  |  |
| 10 | Mar.05 – Mar.11 | **13.57** | **-0.08** | **7.8** | **4.6** | 7 | Feb.10 -Feb.16 | **11** | **-2.2** | **0** | **14** |  |
|  |  |  |  |  |  |  |  |  |  |  |  |  |
| 11 | Mar.12 – Mar.18 | **19.79** | **2.2** | **30.6** | **2.4** | 8 | Feb.17 -Feb.23 | **7.4** | **-0.9** | **5.2** | **11.6** |  |
|  |  |  |  |  |  |  |  |  |  |  |  |  |
| 12 | Mar.19 – Mar.25 | **23.64** | **4.71** | **3.6** | **5.41** | 9 | Feb.24 -Mar.02 | **9.3** | **-0.1** | **3.6** | **13.4** |  |
|  |  |  |  |  |  |  |  |  |  |  |  |  |
| 13 | Mar.26 – Apr.01 | **20.93** | **4.35** | **0** | **8** | 10 | Mar. 03-Mar.09 | **14.3** | **2.9** | **1.8** | **15.5** |  |
|  |  |  |  |  |  |  |  |  |  |  |  |  |
| 14 | Apr.02 – Apr.08 | **20.79** | **8.42** | **11.4** | **4.34** | 11 | Mar.10 – Mar16 | **21.5** | **5.7** | **0.1** | **19.3** |  |
|  |  |  |  |  |  |  |  |  |  |  |  |  |
| 15 | Apr.09 – Apr.15 | **22.5** | **7.14** | **7.4** | **5.25** | 12 | Mar.17 –Mar.23 | **17.6** | **5.7** | **2.5** | **17.5** |  |
|  |  |  |  |  |  |  |  |  |  |  |  |  |
| 16 | Apr. 16-Apr.22 | **22.52** | **8.18** | **30.2** | **6.27** | 13 | Mar. 24–Mar.30 | **23** | **6** | **0.1** | **22** |  |
|  |  |  |  |  |  |  |  |  |  |  |  |  |
|  |  |  |  |  |  | 14 | Mar.31 – Apr.06 | **23.5** | **7.8** | **0.3** | **22** |  |
|  |  |  |  |  |  |  |  |  |  |  |  |  |
|  |  |  |  |  |  | 15 | Apr.07 – Apr. 13 | **21.3** | **7.7** | **1.8** | **21.1** |  |
|  |  |  |  |  |  |  |  |  |  |  |  |  |
|  |  |  |  |  |  | 16 | Apr. 14– Apr. 20 | **18.6** | **8.2** | **2.6** | **18.2** |  |
|  |  |  |  |  |  |  |  |  |  |  |  |  |
|  |  |  |  |  |  | 17 | Apr. 21– Apr. 27 | **20.5** | **8.7** | **3** | **21** |  |
